# The discovery of an overseen pygmy backswimmer in Europe (Heteroptera, Nepomorpha, Pleidae)

**DOI:** 10.1038/s41598-024-78224-6

**Published:** 2024-11-15

**Authors:** Michael J. Raupach, Nele Charzinski, Adrian Villastrigo, Martin M. Gossner, Rolf Niedringhaus, Peter Schäfer, Sebastian Schmelzle, Gerhard Strauß, Lars Hendrich

**Affiliations:** 1 Bavarian State Collection of Zoology, Münchhausenstraße 21, 81247, Munich, Germany; 2 Carl von Ossietzky University Oldenburg, Carl von Ossietzky Straße 9-11, 26129, Oldenburg, Germany; 3 Swiss Federal Institute for Forest, Snow and Landscape Research, Zürcherstraße 111, 8903 Birmensdorf, Switzerland; 4 ETH Zurich, Rämistraße 101, 8092 Zurich, Switzerland; 5 TU Darmstadt, Schnittspahnstraße 3, 64287 Darmstadt, Germany

**Keywords:** 3D scans, DNA barcoding, Illumina, Integrative taxonomy, Mitochondrial genomes, Phylogenomics, Ecology, Evolution, Zoology

## Abstract

The Pleidae, or pygmy backswimmers, is a family of aquatic bugs (Hemiptera, Heteroptera, Nepomorpha) containing four genera. Here, we describe *Plea cryptica* sp. nov. and redescribe its sister species, *Plea minutissima* Leach, 1817. Whereas the morphological distinction of these closely related species is only possible for males, molecular data clearly separate them. As part of our taxonomic study, we provide comprehensive molecular data including more than 200 DNA barcodes from all over Europe, complete nuclear ribosomal DNA, full mitochondrial genome data, and 3D scans for both species. Furthermore, the same molecular markers are also presented for *Neoplea striola* (Fieber, 1844). We used Maximum Likelihood (ML) analyses to reconstruct the phylogeny of the Pleidae and Notonectoidea based on available mitogenomic data. Our study represents a successful implementation of the proposed concept of taxonomics, using data from high-throughput sequencing technologies for integrative taxonomic studies, and allowing high confidence for both biodiversity and ecological research.

**Supplementary Information:**

The online version contains supplementary material available at 10.1038/s41598-024-78224-6.

## Introduction

The Pleidae, commonly known as pygmy backswimmers, is a family of the Nepomorpha, or water bugs (Hemiptera, Heteroptera), with around 40 known species. With a body length between 1.5 and 3.0 mm, they look like tiny backswimmers (Nepomorpha, Notonectidae) but lack their oarlike hind legs^1^. Characterized by their compact, globular bodies and broad, short heads, pygmy backswimmers exhibit a unique anatomical feature: their forewings are elevated laterally and roof-shaped, contributing to their characteristic silhouette. Like the Notonectidae, the Pleidae also swim upside down, rowing with their legs^1^.

Pygmy backswimmers are typical inhabitants of stagnant waters with rich vegetation and climb on aquatic plants^2^, preying upon small arthropods such as mosquito larvae, ostracods, and cladocerans^1,2^. The classification of the four recognized genera of the Pleidae (*Heteroplea* Cook, 2011^3^, *Neoplea* Esaki & China, 1928^4^, *Paraplea* Esaki & China, 1928^4^, and *Plea* Leach, 1817^5^) is based on the tarsal formula^3^. Both *Plea* (one species) and *Heteroplea *(4 species) are characterized by a 3-3-3 tarsal formula^6,7^. The genus *Heteroplea*, however, can be distinguished from *Plea* in having a callus of thickened cuticle on the vertex of the head and an ovipositor that lacks spurs on its sides, along with other minor differences^3^. In addition, both taxa are separated geographically: whereas *Plea* is documented from Europe, North Africa, and Asia, species of the genus *Heteroplea* are restricted to South America^3^. In contrast, *Neoplea*, comprising 17 species, features a 3-2-3 tarsal formula^3,8^ whereas the 21 known species of the genus *Paraplea* are characterized by a 3-2-2 tarsal formula^3,7,9^. So far, the phylogenetic history of the different genera as well as species within the Pleidae is unknown. Various studies, however, found evidence for a close relationship between the pygmy backswimmers and the Helotrephidae^10–12^.

Pygmy backswimmers are broadly distributed but show their greatest diversity in the tropics (e.g., South America, Asia). *Plea minutissima* Leach, 1817^5^ is the only species recorded from Europe, with two described subspecies: *P. m. minutissima* Leach, 1817^5^ and *P. m. tassilii* Poisson, 1953^13^. Whereas *P. m. minutissima* is widespread and abundant across the Western Palaearctic region^2,14,15^ with an extension to Central Asia including Iraq^16^, Turkey^17^, and Kazakhstan^18^, *P. m. tassilii* is only known from southeastern Algeria^13^. The taxonomic history of *P. minutissima* is characterized by the erection of various synonyms in the past. The original description was made by Leach (1817)^5^, based on animals collected “*in aquis stagnantibus prope Londinum vulgatissime*” (“most common in stagnant waters near London”). Subsequently, Rey (1894)^19^ delineated the subspecies *P. m. sublaevis* Rey, 1894, sourced from Provence, France. Another taxonomic addition came in the form of *P. leachi* McGregor & Kirkaldy, 1899, described from specimens collected in Perthshire, Scotland^20^. Following meticulous examinations, Kerzhner (1978)^21^ synonymized *P. leachi* and *P. m. sublaevis* under *P. m. minutissima*, establishing the last one as the only valid species in the genus *Plea*. Kerzhner, however, ignored *P. m. tassilii* in his study. It should be also noted that another name, *Notonecta atomaria* Pallas, 1771^22^, was sometimes regarded as a synonym of *P. minutissima* in the past as well. This classification, however, is based on a misidentification, as it corresponds to *Notonecta minutissima* Linnaeus, 1758^23^, which is now recognized as the water boatman species *Micronecta minutissima* (Linnaeus, 1758) (Nepomorpha, Corixidae)^21^.

The biology of *P. minutissima *has been intensively studied in the past, clarifying various aspects of the life history of this water bug^24–27^. For example, it is known that the reproductive cycle starts in early summer, with eggs laid and embedded within aquatic plant leaves. About three weeks later, larvae emerge, and the postembryonic development lasts about one and a half months. Molting from larvae to imago typically occurs from August until the end of September. During hibernation, which occurs in the imaginal stage, individuals settle at the water’s bottom, remaining motionless for months and alternating between bubble and plastron respiration^25^. Since *P. minutissima* can hibernate twice, they are able to pass through two reproductive cycles, each in the early summer. Similar to other water bugs^28–31^, *P. minutissima* can communicate under water via stridulation, likely facilitating swarm formation and mate selection^24,32,33^. Stridulation involves a nodding movement of the fused head and prothorax, with a chitinous protrusion of the prothorax rubbing against the stridulatory surface of the mesothorax. Both sexes possess the ability to stridulate and the possession of a tympanic organ, although sonograms remain undocumented so far.

As part of a previous DNA barcoding survey of the water bugs of Germany, two distinct molecular clusters with genetic distances ranging from 8.1 to 8.3% were found within *P. m. minutissima*, pointing to the existence of cryptic species in Europe^34^. Here, we describe an overseen species in the genus *Plea*, *P. cryptica* sp. nov., and redescribe *P. m. minutissima*. Both species descriptions are enriched with comprehensive morphological data, 3D scans, DNA barcodes, mitochondrial genomes, and complete nuclear 18S and 28S rRNA gene sequences. In addition, we provide full mitochondrial genome data and nuclear rRNA sequence data for another species of the Pleidae, *Neoplea striola* (Fieber, 1844)^35^, for comparison. This comprehensive approach to species descriptions aligns with the proposed concept of “taxonomics”, advocating for the utilization of intricate data derived from modern molecular technologies in taxonomic studies^36^.

## Results

### Molecular data: DNA barcoding

**Figure 1:**
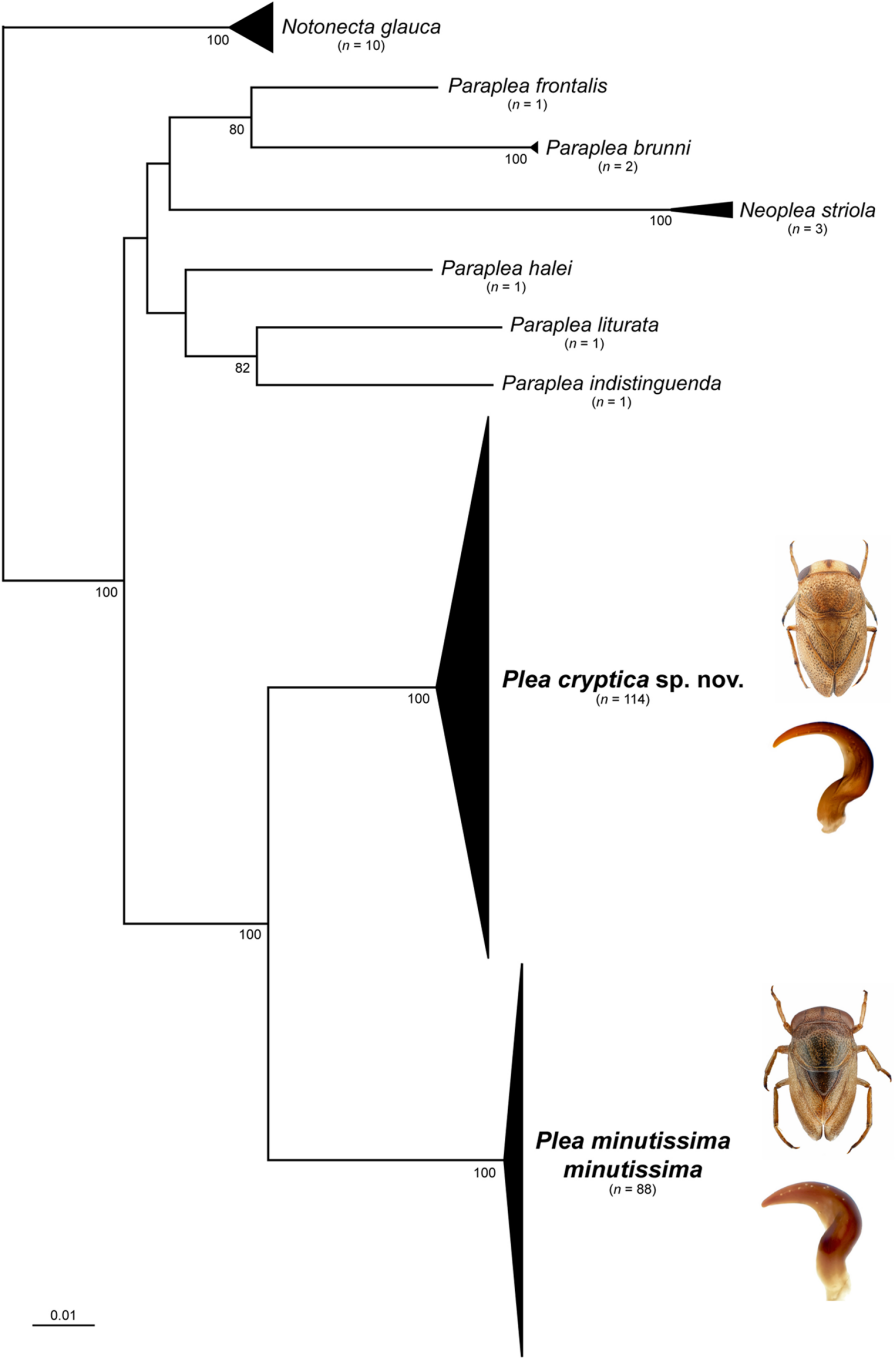
Neighbor-joining (NJ) topology of the analyzed Pleidae DNA barcodes based on Kimura 2-parameter distances. Triangles show the relative number of specimens sampled (height) and sequence divergence (width). Numbers next to nodes represent non-parametric bootstrap values > 80% (1,000 replicates).

Overall, 202 DNA barcode sequences were analyzed, with fragment lengths ranging from 403 to 658 bp. The analysis revealed two clusters, the first one with 88 sequences (*P. m. minutissima*) and the second (*P. cryptica* sp. nov.) with 114 sequences, representing distinct species (see species description below). Consistent with the typical AT-rich peculiarity of arthropod mitochondrial genomes, the DNA barcode region of the Pleidae exhibited a high AT-content, with a mean sequence composition of A = 0.34, C = 0.17, G = 0.16, and T = 0.33. Intraspecific K2P distances ranged from 0 to a maximum of 2.63% for *P. m. minutissima*, and 1.96% for *P. cryptica* sp. nov. No phylogeographic patterns were observed within both species. Interspecific distances between the analyzed species ranged from 6.56% (*P. cryptica* sp. nov./*P. m. minutissima*) and 12.5% (*Neoplea striola*/*Paraplea halei*). A detailed summary of all distances and BINs is shown in Table 1. The NJ analyses based on K2P distances revealed non-overlapping clusters with bootstrap support values > 95% for all species with more than one analyzed specimen (Fig. 1).

**Table 1:** Molecular distances based on the Kimura 2-parameter model of the analyzed DNA barcodes of the Pleidae.

Species	*n*	BINs	Mean ISD	Max ISD	NN	Distance to NN
*Notonecta glauca*	10	AAK4442	0.48	1.4	*Paraplea indistinguenda*	9.78
*Neoplea striola*	3	ACE0078	1.13	1.59	*Paraplea halei*	12.5
*Paraplea brunni*	2	ADV7904	0.15	0.15	*Paraplea frontalis*	7.45
*Paraplea frontalis*	1	ADC2910	n.a.	0	*Paraplea brunni*	7.45
*Paraplea halei*	1	AAZ9975	n.a.	0	*Paraplea indistinguenda*	8.55
*Paraplea indistinguenda*	1	AEU9425	n.a.	0	*Paraplea liturata*	7.58
*Paraplea liturata*	1	AEU9426	n.a.	0	*Paraplea indistinguenda*	7.58
*Plea cryptica* sp. nov.	114	AAF3832	0.24	1.96	*Plea m. minutissima*	6.56
*Plea m. minutissima*	88	ACY0868	0.35	2.63	*Plea cryptica* sp. nov.	6.56

Divergence values were calculated for all studied sequences, using the Nearest Neighbour Summary implemented in the Barcode Gap Analysis tool provided by the Barcode of Life Data System (BOLD). Align sequencing option: BOLD aligner (amino acid based HMM), ambiguous base/gap handling: pairwise deletion. Barcode Index Numbers (BINs) are based on the barcode analysis from 15-12-2023. With *n* = number of specimens, ISD = intraspecific distance, and NN = nearest neighbor.

### Molecular data: complete 18S and 28S rRNA genes

Seven sequences of the complete nuclear 18S rRNA gene were generated for the studied *Plea* species (*P. m*. *minutissima*: *n* = 3, *P. cryptica* sp. nov.: *n* = 3, *N. striola*: *n* = 1). No intragenomic or intraspecific variations were detected. The complete 18S rRNA sequences had a total length of 1,902 bp for both *Plea* species (*N. striola*: 1,901 bp). The average base frequencies were A = 0.25, C = 0.23, G = 0.27, and T = 0.25 (*N. striola*: A = 0.25, C = 0.23, G = 0.27, T = 0.25). The sequences of both *Plea* species had a patristic distance of 0.1%, based on two transversions (Table 2).

**Table 2:** Patristic pairwise distances of complete 18S rRNA gene sequences of *Neoplea striola* (Fieber, 1844), *Paraplea frontalis*, *Plea m. minutissima*, and *Plea cryptica* sp. nov. (lower triangle).

	*Neoplea striola* (*n* = 1)	*Paraplea frontalis* (*n* = 1)	*Plea m. minutissima* (*n* = 3)	*Plea cryptica* sp. nov. (*n* = 3)
***Neoplea striola*** **(*n*** **= 1)**	-	38 / 20 / 8	43 / 19 / 8	43 / 17 / 8
***Paraplea frontalis*** **(*n*** **= 1)**	0.0305	-	11 / 5 / 2	11 / 3 / 2
***Plea m. minutissima*** **(*n*** **= 3)**	0.0327	0.0074	-	0 / 2 / 0
***Plea cryptica*** **sp. nov**. **(*n*** **= 3)**	0.0316	0.0063	0.0011	-

Total alignment length = 1,907 base pairs. Upper triangle: Number of observed transitions (left), transversions (central), and insertions/deletions (right). With *n* = number of analyzed specimens.

In the case of the complete 28S rRNA sequences, both *Plea* sequences had a length of 4,075 bp (*N. striola*: 3,869 bp); the average base frequencies were A = 0.22, C = 0.26, G = 0.31, and T = 0.21 (*N. striola*: A = 0.22, C = 0.26, G = 0.31, T = 0.21). Patristic distances between both sequences of the genus *Plea* had a value of 0.07%, with two transitions and one transversion (Table 3).

**Table 3:** Patristic pairwise distances of complete 28S rRNA gene sequences of *Neoplea striola* (Fieber, 1844), *Paraplea frontalis* (Fieber, 1844), *Plea m. minutissima* Leach, 1817, and *Plea cryptica* sp. nov. (lower triangle).

	*Neoplea striola* (*n* = 1)	*Paraplea frontalis* (*n* = 1)	*Plea m. minutissima* (*n* = 1)	*Plea cryptica* sp. nov. (*n* = 1)
***Neoplea striola*** **(*n*** **= 1)**	-	123 / 90 / 14	135 / 89 / 20	135 / 90 / 20
***Paraplea frontalis*** **(*n*** **= 1)**	0.0552	-	42 / 19 / 3	44 / 19 / 3
***Plea m. minutissima*** **(*n*** **= 1)**	0.0581	0.0158	-	2 / 1 / 0
***Plea crytica*** **sp. nov.** **(*n*** **= 1)**	0.0583	0.0166	0.0007	-

Total alignment length = 4,090 base pairs. Upper triangle: Number of observed transitions (left), transversions (central) and insertions/deletions (right). With *n* = number of analyzed specimens.

### Molecular data: high-throughput sequencing output and mitochondrial genome reconstruction

A total of 5,896,894 paired reads were generated for *P. m. minutissima*, 8,860,394 for *P. cryptica* sp. nov. and 8,246,826 for *N. striola*. The mean base coverage of the mitochondrial contig was 293-fold for *P. m. minutissima*, 351-fold for *P. cryptica* sp. nov., and 186-fold for *N. striola*. The complete mitochondrial genomes of *P. m. minutissima* was 15,338 bp in length, that of *P. cryptica* sp. nov. 15,624 bp, whereas the mitochondrial genome of *N. striola* had a length of only 14,376 bp.

**Figure 2:**
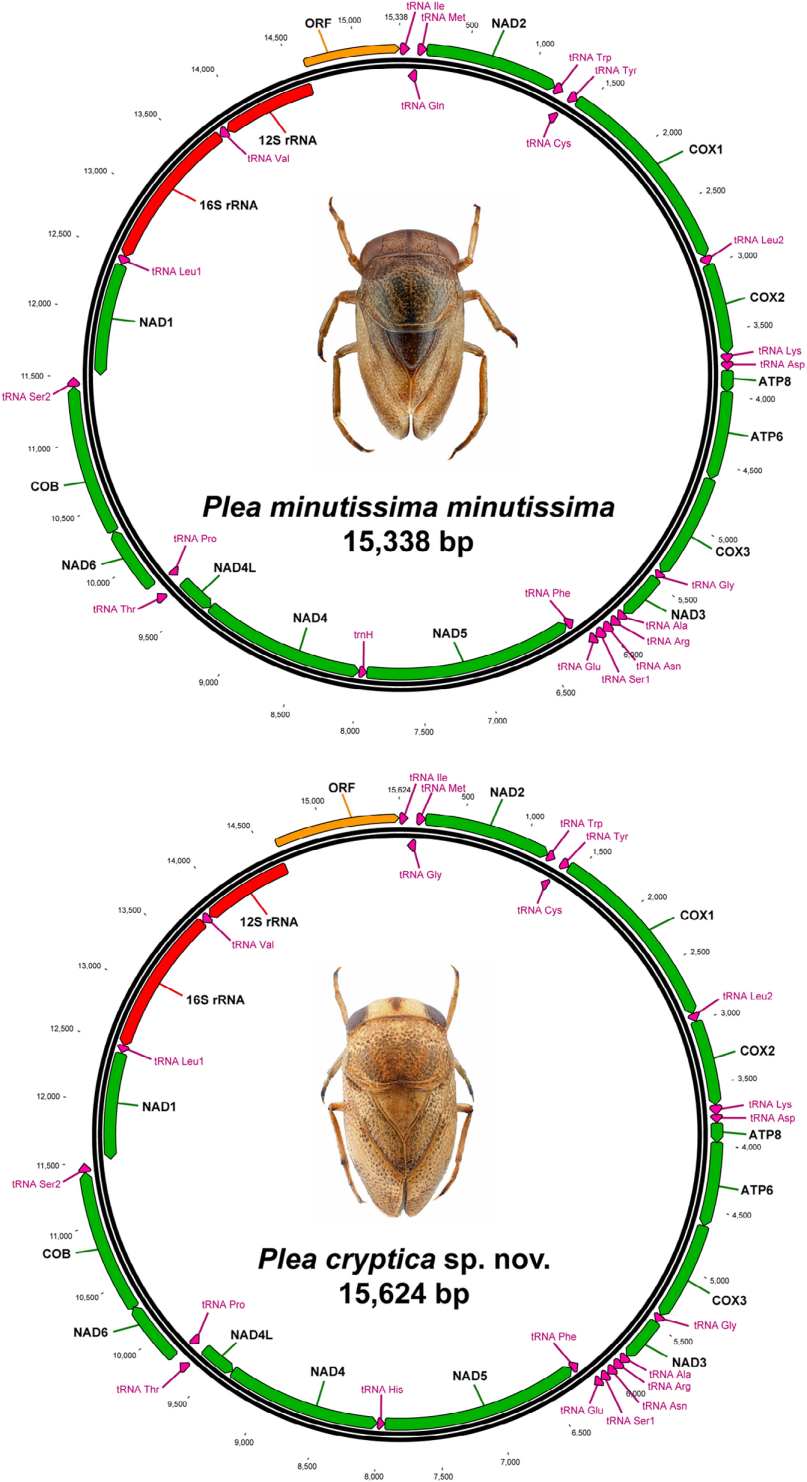
Organization of the mitochondrial genome of *Plea m. minutissima* Leach, 1817 and *Plea cryptica* sp. nov. Protein encoding genes are marked in green, ribosomal RNAs in red, tRNAs in violet, and the putative control region in orange. Abbreviations: ATP6/8: ATPase subunits 6/8; COX1-3: Cytochrome *c* oxidase subunits 1–3; NADH1-6/4 L: NADH dehydrogenase subunits 1–6/4 L, and COB: Cytochrome *b*. All 22 transfer RNA genes are designated by tRNA-X and labelled according to the IUPAC-IUB single-letter amino acid codes. Arrowheads indicate the direction of transcription.

In general, both mitochondrial genomes of the genus *Plea* were very similar. They consisted of a single circular DNA molecule that carried 13 protein-coding genes, two subunits of the mitochondrial RNA, 22 tRNAs, one for each amino acid except leucine and serine, each of which had two copies (tRNA^Leu1^/tRNA^Leu2^, tRNA^Ser1^/tRNA^Ser2^), and a putative control region (Fig. 2, Supplementary Table S1). The gene organizations of both genomes were identical to the basic pattern of insects. For both species, the heavy strands (H-strand) encoded 23 genes (protein-coding genes: 9, tRNAs: 14), whereas the remaining 14 genes were located on the light strands (L-strand). Twelve gene overlaps and 6 intergenic spacers were revealed for the genome of *P. m. minutissima*, as well as 13 gene overlaps and 6 intergenic spacers for the genome of *P. cryptica* sp. nov.

For *P. m. minutissima*, the total length of overlaps was 38 bp, ranging from 1 to 11 bp, whereas intergenic spacers had values from 1 to 17 bp, resulting in a total length of 25 bp. For *P. cryptica* sp. nov., the total number of overlaps was 39 bp with values between 1 and 11 bp, and 25 bp (1 to 17 bp) from intergenic spacers. Furthermore, we found similar nucleotide compositions for both genomes, with frequencies of A = 0.45, C = 0.12, G = 0.10, and T = 0.33 for *P. m. minutissima* and A = 0.44, C = 0.14, G = 0.11, and T = 0.31 for *P. cryptica* sp. nov. The gene arrangement of the mitochondrial genome of *N. striola* was identical to those of the two *Plea* species (Supplementary Table S2); ten gene overlaps with a total length of 29 bp and values ranging from 1 to 8 bp were found. Furthermore, 8 intergenic spacers with a total number of 35 bp were detected, with values between 1 and 20 bp. Frequencies of the nucleotides for the complete genome were A = 0.45, C = 0.13, G = 0.09, and T = 0.33.

In total, 13 protein-coding genes (PCGs) were identified for both *Plea* genomes, with a total length of 11,101 bp, which encode 3,698 amino acid (aa) residues (*N. striola*: 11,075 bp, 3,689 aa). For all genomes, nine PCGs were encoded from the H-strand (COB, COX1, COX2, COX3, ATP6, ATP8, NAD2, NAD3, NAD6) and four from the L-strand (NAD1, NAD4, NAD4L, NAD5). All protein-coding genes had typical invertebrate mitochondrial start codons (ATA, ATG, or ATT), but some of them displayed incomplete stop codon (single T or TA) as known from other arthropod species. P-distances between the aligned *Plea* PCGs ranged from 6.7% (NAD1) to 10.9% (ATP8) (Supplementary Table S1).

Furthermore, a total of 22 tRNA genes with lengths from 59 bp (tRNA^Tyr^) to 71 bp (tRNA^Lys^, tRNA^Trp^, tRNA^Val^) were found for both *Plea* genomes (Supplementary Table S1) (*N. striola*: tRNA^Ala^: 62 bp, tRNA^Lys^: 72 bp). Most tRNAs (*n* = 14) were encoded on the H-strand, whereas the remaining eight tRNAs were located on the L-strand. Both rRNA genes were found on the L-strands of the three genomes, with lengths of 777 bp for the 12S rRNA gene and 1,269 bp for the 16S rRNA gene of *P. m. minutissima*. For *P. cryptica* sp. nov., the 12S rRNA gene had a length of 786 bp whereas the length of the 16S rRNA gene was 1,272 bp (*N. striola*: 12S rRNA: 760 bp, 16S rRNA: 1,265 bp). The p-distance was 4.5% for the aligned 12S rRNA genes and 5.5% for the 16S rRNA genes (Supplementary Table S1).

The major non-coding regions (open reading frame, ORF) were identified for all genomes between the 12S rRNA and tRNA^Ile^ genes, which are considered to include the putative control region (Supplementary Table S1). The length of this major ORF was 726 bp for *P. m. minutissima* and 995 bp for *P. cryptica* sp. nov. (*N. striola*: 714 bp). All other non-coding regions were much smaller, ranging from 1 to 17 bp for both species (*N. striola*: 1 to 20 bp). The A + T contents of the ORFs were higher than those of other mitogenome regions, with 0.75 for *P. m. minutissima* and 0.79 for *P. cryptica* sp. nov. (*N. striola*: 0.68). Furthermore, both ORFs consisted of several different repetitive motifs in tandem (e.g., (TCCC)_n_ or (TA)_n_).

### Molecular data: phylogenetic analysis of the mitochondrial genomes

**Figure 3:**
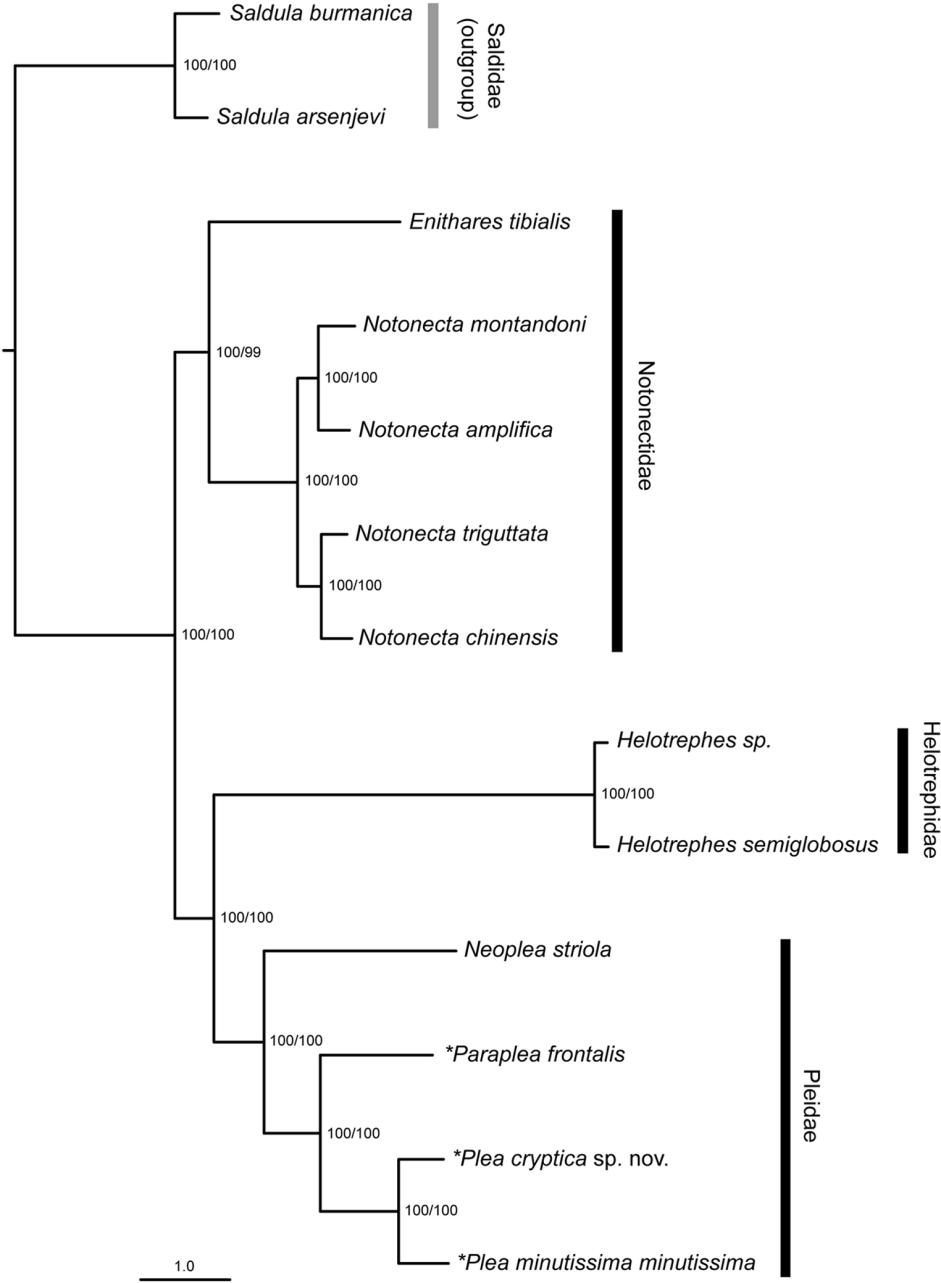
Maximum Likelihood phylogeny of the Notonectoidea based on mitochondrial genome data inferred in IQ-TREE. The different models of nucleotide substitution used for each gene were selected with Modelfinder as part of the IQ-TREE work package. The topology was rooted with three specimens of the Saldidae as outgroup. Asterisks indicate newly sequenced mitochondrial genomes. Numbers next to nodes represent UFBoot (left) and SH-aLRT (right) values (see Material and Methods for more details).

All species of the monophyletic Notonectoidea were divided up into two clades, with maximum supporting values (UFBoot: 100, SH-aLRT: 100) for almost all nodes (Fig. 3). The first clade included all five species of the Notonectidae, with *Enithares tibialis* Liu & Zheng, 1991^37^ as sister taxon to the four studied species of the genus *Notonecta*. These in turn were divided into two clades, with *Notonecta montandoni* Kirkaldy, 1897^38^ and *N. amplifica* Kiritshenko, 1930^39^, as well as *N. triguttata *Motschulsky, 1861^40^ and *N. chinensis* Fallou, 1887^41^ as species pairs. The second clade included all species of the Helotrephidae and Pleidae, which were found as sister taxa to each other. Within the Pleidae, *N. striola* is the sister taxon to *P. frontalis* and the two *Plea* species.

### Systematics

**Genus *Plea*** **Leach**,** 1817**^5^.

*Plea* Leach, 1817: 11^5^. Type species by monotypy: *Plea minutissima* Leach, 1817^5^.

*Ploa* Stephens, 1829a: 354^42^; 1829b: 66^43^. Incorrect subsequent spelling.

*Ploa* Burmeister, 1835: 189^44^. Unjustified emendation.

*Ploea* Douglas & Scott, 1876: 61^45^. Unjustified emendation.

***Plea minutissima minutissima*** **Leach**,** 1817**^5^.

*Plea minutissima* Leach, 1817: 14^5^.

*Plea minutissima* var. *sublaevis* Rey, 1894: 13^19^ (syn. Kerzhner, 1977: 357)^21^.

*Plea leachi* McGregor & Kirkaldy, 1899: 4^20^. For *Notonecta minutissima *auct., non Linnaeus, 1758^23^.

*Plea atomaria* (non Pallas, 1771)^22^: Horvath, 1918a: 336^46^. Misidentification: Kerzhner, 1977: 57^21^.

*Plea minutissima minutissima* Leach, 1817: Stichel, 1955-56: 102^47^.

### Type material examined

**Syntype(s)** lost; locality: London, United Kingdom (“*habitat in aquis stagnantibus prope Londinum vulgatissime*”).

Leach’s (1817)^5^ types are supposed to be in the Natural History Museum, London, United Kingdom (NHM). However, it is known that (at least) his Corixidae material was lost at some point between 1937 and 1955^48^. According to the Hemiptera curator at the NHM, Diana Isabel Rendón-Mera, the type material of *Plea minutissima* is also lost. Therefore, we decided to designate a neotype from material freshly collected in England and identified based on morphological and molecular data.

**Neotype**: adult male, 2.3 mm, “England, Somerset, Sedgemoor, Somerset Levels, Pawlett Hams, N 51.179222 E -3.024722, 09-Oct-2021, coll. D. Bilton”, “ZSM_HET_1144”.

The neotype, which will be deposited in the NMH, is provided with a red printed label. It is designated to support taxonomic stability, as there is a morphological similar sister species, widely distributed in central and eastern continental Europe, and it is therefore paramount for present and future investigations to possess unambiguously characterized name bearing specimens for all European species of the genus.

### Additional material examined

Further material examined included 83 adult specimens, with 64 males and 19 females.

**Armenia**: 1 specim., “Armenia, Tavush, Zovk, 19-Jun-2007, N 40.068611 E 45.000556, leg. M. Kalashian”, “ZSM_HET_1127”.

**Belgium**: 2 specim., “Belgium, Corsendonk, Tikkebroeken, 09-Jun-2017, N 51.4841667 E 5.218056, leg. Lars Hendrich”, “ZSM_HET_0840”, idem, “ZSM_HET_0841”; 18 specim., “Belgium, East Flanders, Sint-Niklaas, Beveren, 09-Jun-2017, N 51.4377778 E 4.386389, leg. Lars Hendrich”, “ZSM_HET_0844” idem, “ZSM_HET_0845–0850”, “ZSM_HET_0881–0888”, “ZSM_HET_0895”, “ZSM_HET_0896”, ”ZSM_HET_0914”.

**France:** 2 specim., “France, Gironde, Nouvelle-Aquitaine, Arcachon, La Teste, 22-28-Aug-1956, N 44.658611 E -1.168889, leg. Weber”, “ZSM_HET_1514”, idem, “ZSM_HET_1516” ; 2 specim., “France, Pyrénées Orientales, Banyuls-sur-Mer, 27-Jul-11-Aug-1956, N 42.482222 E 3.1275, leg. Weber”, “ZSM_HET_1506” idem, “ZSM_HET_1507”; 3 specim., “France, Provence-Alpes-Côte d’Azur, Bouches-du-Rhône, Saintes-Maries-de-la-Mer, 04-Jul-1977, N 43.459167 E 4.420278, leg. E.-G. Burmeister”, “ZSM_HET_1365–1367”; 3 specim., “France, Bretagne, Finistère, Brest, Saint-Renan, 06-Jul-1980, leg. E.-G. Burmeister”, “ZSM_HET_1368–1370”.

**Germany:** 1 specim., “Germany, Baden-Württemberg, Möggingen, Mindelsee, N 47.754444 E 9.022222, leg. Ralf Heckmann”, “ZSM_HET_1361”; 3 specim., “Germany, Lower Saxony, Ammerland, Jaderberg, 03-Jun-2016 N 53.334533 E 8.185283, leg. Nadine Lange”, “ZSM_HET_0819”, idem, “ZSM_HET_0824”, “ZSM_HET_0830” ; 9 specim., “Germany, Lower Saxony, Schwanewede, Harrier Sand, 14-Sep-2016, N 53.309271 E 8.499151, leg. Rolf Niedringhaus”, “ZSM_HET_0731”, idem, “ZSM_HET_0732–0739”; 1 specim., “Germany North Rhine-Westphalia, Landkreis Steinfurt, Recke, Heiliges Meer, 03-Apr-2017, N 52.351944 E 7.383611, leg. Rolf Niedringhaus”, “ZSM_HET_0758”; 1 specim., “Germany, North Rhine-Westphalia, Münsterland, Münster, N 51.962944 E 7.628694, leg. Rolf Niedringhaus”, “ZSM_HET_0880”; 4 specim., “Germany, Nordrhein-Westfalen, Münsterland, Haltern am See, Sandabgrabung Quarzwerke, 12-Aug-2020, N 51.713119 E 7.255204, leg. Michael Raupach”, “ZSM_HET_1186”, idem, “ZSM_HET_1358–1360”.

**Italy:** 2 specim., “Italy, Sardinia, Oristano, Giara di Gesturi, 10-Aug-1978, N 39.746944 E 8.944167, leg. E.-G. Burmeister”, “ZSM_HET_1376–1377”.

**Morocco:** 1 specim., “Morocco, Ifrane, Fez, 11-Jun-2007, N 33.575833 E -4.9775, leg. Aguilera Hernando & I. Ribera”, “ZSM_HET_1121”.

**Spain:** 5 specim., “Spain, Andalusia, Huelva, 26-Jun-1977, N 37.25 E -6.95, leg. Carlos Montes”, “ZSM_HET_0859”, idem, “ZSM_HET_0860”, “ZSM_HET_0861”, “ZSM_HET_0863”, “ZSM_HET_0864”; 4 specim., “Spain, Balearic Islands, Menorca, 01-Aug-1983, N 39.970556, E 4.081667, leg. Carlos Montes”, “ZSM_HET_0878”, idem, “ZSM_HET_0879”, “ZSM_HET_0910”, “ZSM_HET_0911”; 1 specim., “Spain, Catalunya, Barcelona, 11-15-Jul-1959, N 41.4 E 2.166667, leg. Weber”, “ZSM_HET_1525”.

**Tunisia:** 1 specim., “Tunisia, Kairouan, Queslatia, 24-Oct-2001, N 35.84 E 9.58, leg. I. Ribeira & A. Cieslak”, “ZSM_HET_1132”.

**Turkey:** 1 specim., “Turkey, Sivas, Sivas, Demiryurt, Tödürge Gölü, 16-May-2005, N 39.875278 E 37.610556, leg. P. Kment”, “ZSM_HET_1117”.

**United Kingdom** (England): 5 specim., “England, Somerset, Sedgemoor, Somerset Levels, Pawlett Hams, 09-Oct-2021, N 51.179722 E -3.024722, leg. David Bilton”, “ZSM_HET_1141”, idem, “ZSM_HET_1143”, “ZSM_HET_1145”, “ZSM_HET_1146”, ”ZSM_HET_1147”.

All specimens are deposited in the SNSB-ZSM.

### Description

All measurements are given in mm.

### Body size

Body length 2.10–2.60; average 2.36 (neotype: 2.30). Body length 1.80–3.00 has been documented^49–52^. See Supplementary Movie S1 for a movie of a representative specimen.

### Color

**Figure 4:**
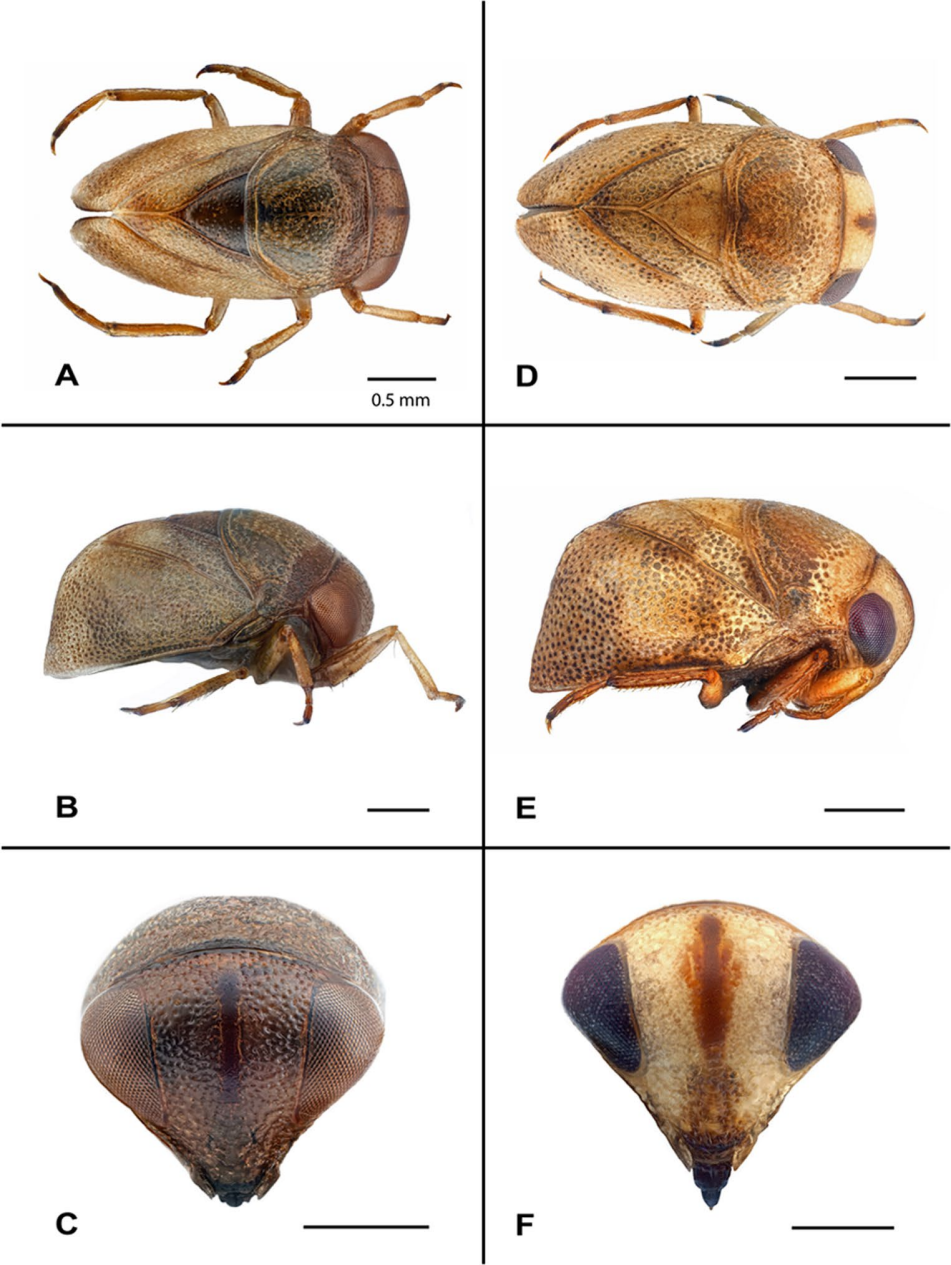
Male specimen of *Plea m. minutissima* Leach, 1817 from France, Pyrénées Orientales, Banyuls-sur-Mer. **(A)** Dorsal view of a specimen with irregular honeycomb structure, dark brown scutellum and pronotum. **(B)** Lateral view of the same specimen with irregular honeycomb structure. **(C)** Frontal view of the same specimen with distinct dark brown bar between the eyes. Male specimen of *Plea cryptica* sp. nov. from Germany, Berlin, Zehlendorf, Wannsee. **(D)** Dorsal view with irregular honeycomb structure, light brown scutellum and pronotum. **(E)** Lateral view of the same specimen with irregular honeycomb structure. **(F)** Frontal view of the same specimen with diffuse light brown bar between the eyes. Scale bar = 0.5 mm.

Color quite variable even among individuals within a population; base color from almost white, bright yellow over light brown to dark brown; with irregular honeycomb-like indentations with setae at bases of pits, especially on pronotum; scutellum usually a bit lighter than wings and pronotum; legs yellowish-brown, distal and proximal ends usually darker; ventral side usually darker than dorsal side; eyes dark red to black; vertical light brown to dark brown bar between eyes (Fig. 4A–C). For the neotype, base color is beige.

### Head

Head generally concolorous with body, sometimes more intense; mouthparts usually dark brown; bar can differ between individuals in intensity, acuity, and coloration, ranging from narrow, blurred light brown bars to broad, definite dark brown bars (for the neotype, a blurred light brown bar is present); antenna three-segmented, usually visible below eye; interocular distance from edge towards vertex 0.67–0.77 (neotype: 0.71), from edge towards mouthparts 0.49–0.57 (neotype: 0.52).

### Pronotum

Base color ranging cream to light brown (neotype: amber); irregular honeycombing apparent on most specimens; number, size, and shape of indentations highly variable; most punctures with setae at bases.

### Wings

Complete to posterior end of body; punctures (0.02 in diameter) evenly distributed over entire hemelytra, without defined pattern and underlying irregular honeycomb structure; claval suture distinct, complete; scutellum as long as wide, base color amber-colored to light brown; scutellum amber in the neotype; pits on wings usually with dark base, especially at posterior region; edge of corium usually darker than rest of wing; hindwing membranous, fully developed, completely concealed by hemelytra.

### Legs

Mesothoracic leg shortest, metathoracic leg longest; tibiae pilose; femora sparsely covered with setae, often with single long seta near base; tarsal formula 3-3-3; average leg measurements: prothoracic coxa: 0.05, trochanter: 0.15, femur: 0.65, tibia: 0.60 tarsomere I: 0.03, tarsomere II: 0.10, tarsomere III: 0.15, pretarsal claw: 0.07, total leg length: 1.83; mesothoracic coxa: 0.05, trochanter: 0.10, femur: 0.65, tibia: 0.50, tarsomere I: 0.03, tarsomere II: 0.13, tarsomere III: 0.15, pretarsal claw: 0.10, total leg length: 1.71; metathoracic coxa: 0.05, trochanter: 0.20, femur: 0.73, tibia: 0.70, tarsomere I: 0.05, tarsomere II: 0.25, tarsomere III: 0.28, pretarsal claw: 0.15, total leg length: 2.41.

### Median ventral keel

Starting in an almost rectangular part beneath head, followed by angular prosternal keel with rounded edges and small posterior tooth; mesothoracic keel almost angular but inclined posteriorly; metathoracic keel irregularly shaped, with slot; first abdominal keel fused to metathoracic keel, followed by three almost fused parts with posterior teeth; four distinct teeth associated with first four abdominal segments (see Supplementary Fig. S1A).

### Female characters

Ovipositor roughly triangular; about 0.40 long; width 0.10 at widest point; posterior border (apical row) with 5–10 teeth; surface of ovipositor with 6–11 teeth (see Supplementary Fig. S2); subgenital plate subtriangular; average length 0.55; surface slightly rugose on vaulted basal part, apically followed by series of pits with setae; surface darker on posterior third, sparsely covered with setae, median slot on posterior third.

### Male characters

**Figure 5:**
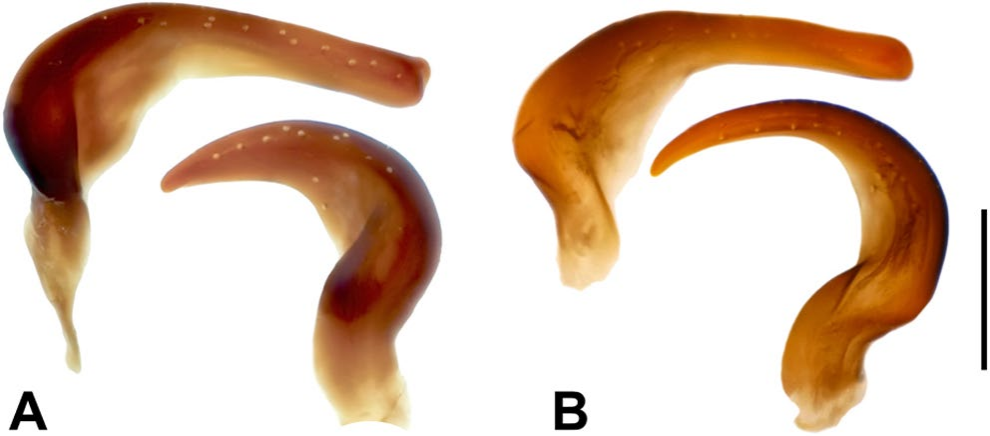
**(A)** Male parameres of *Plea m. minutissima* Leach, 1817 and **(B)*** Plea cryptica* sp. nov. Above: right parameres, below: left parameres. Scale bar = 0.1 mm.

Subgenital plate slightly longer than wide; average length 0.53 (neotype: 0.52); average with 0.47 (neotype: 0.47); without vaulted basal area found in females; surface slightly rugose, with short setae throughout; posterior third with slightly longer setae; right paramere curved at right angles, with rounded tip; average length 0.18 (neotype: 0.18); left paramere compact (Fig. 5A), hook-shaped; average length 0.18 (neotype: 0.17); apical end strongly rounded; base without folding.

### Habitat

**Figure 6:**
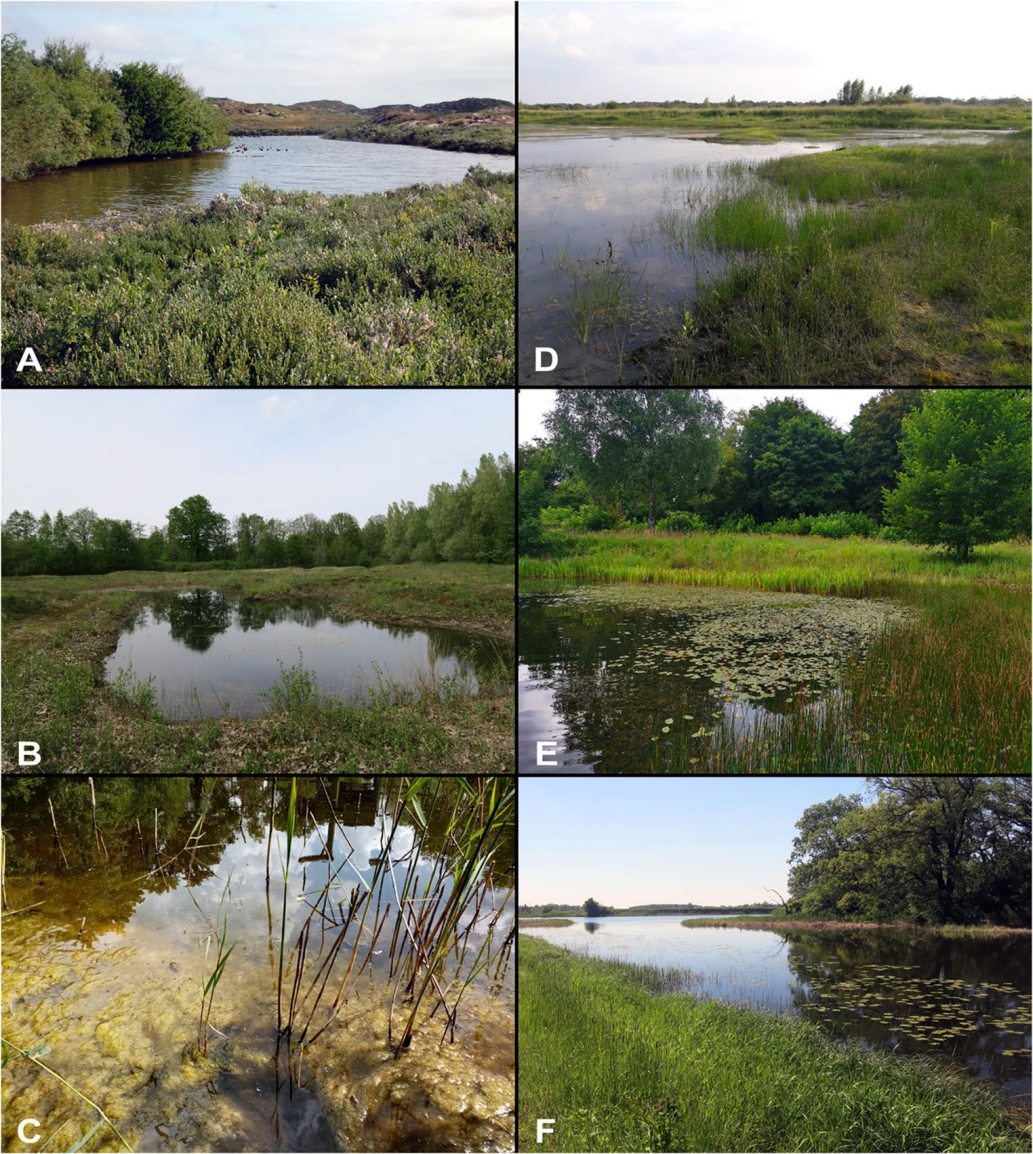
Habitats of *Plea m. minutissima*: **(A)** Pond on Norderney, Lower Saxony, Germany, June 2023. **(B)** Pond near Lingen, Lower Saxony, Germany, May 2006. **(C)** Former fire extinguishing pond in Haltern-Flaesheim, North Rhine Westphalia, Germany, June 2022. Habitats of *Plea cryptica* sp. nov.: **(D)** Oxbow of the river Elbe near Lenzen, Brandenburg, Germany, August 2008. **(E)** Eutrophic pond at Teltow Canal, Berlin-Wannsee, Germany, August 2023. Type locality of *Plea cryptica* sp. nov. **(F)** Emerged vegetation in the oxbow ”Große Streng“, Wartenburg, Sachsen-Anhalt, May 2021.

Eutrophic exposed and more permanent water bodies with fluctuating water level, rich in vegetation with a muddy bottom, such as ponds, kettle holes, fire extinguishing ponds, fens, edges of smaller lakes, stagnant or slow flowing ditches and oxbows (Fig. 6A─C). Adults among emerged and submerged plants, plant debris and mud, often in very shallow water.

### Distribution

Armenia, Belgium, France, Germany, Italy, Morocco, Spain, Tunisia, Turkey, and United Kingdom (England). This species can be expected in other Western countries and regions, e.g., Portugal or Ireland^14^, but has to be confirmed.

### *Plea cryptica* Raupach, Charzinksi and Hendrich, sp. nov.

**ZooBank registration number**: urn:lsid:zoobank.org:pub:BD490C7A-FACB-479E-9CBD-8F2B21CB134E

**Chresonym**: *Plea minutissima minutissima* Leach, 1817, Polhemus, J.T. Pleidae in Catalogue of the Heteroptera of the Palaearctic Region. Volume 1: Enicocephalomorpha, Dipsocoromorpha, Nepomorpha, Gerromorpha and Leptopodomorpha (ed. Aukema, B. & Rieger, C.) 73–75 (The Netherlands Entomological Society, 1995). [in part; countries: Albania, Austria, Croatia, Finland, Germany, Greece, Netherlands, Romania, Switzerland, and the Ukraine.]^14^.

**Type locality:** Germany, Berlin, Zehlendorf, “Pappelteich” at “Teltow Canal” [N 52.397428 E 13.152314].

**Type material examined: Holotype**: Adult male, 2.5 mm, “Germany, Berlin, Zehlendorf, Wannsee, Pappelteich am Teltowkanal, N 52.397428 E 13.152314, 31-Aug-2018, leg. L. Hendrich“, “ZSM_HET_0980“.

**Paratypes:** 69 specimens.

**Germany:** 10 specimens with the same data as holotype but “ZSM_HET_0971“, “ZSM_HET_0972–ZSM_HET_0974”, “ZSM_HET_0976”, “ZSM_HET_0978”, “ZSM_HET_0979”, “ZSM_HET_0981”, “ZSM_HET_0983”, “ZSM_HET_0984”; 1 specim., “Germany, Bavaria, Isartal, Thürnthenning, Königsauer Moos, 01-Jun-2015, N 48.675 E 12.535278, leg. Rolf Niedringhaus“, “ZSM_HET_777“; 1 specim., “Germany, Brandenburg, Cottbus, 08-May-2016, 51.760844 14.327172, leg. Rolf Niedringhaus”, “ZSM_HET_0764”; 53 specim., “Germany, Sachsen-Anhalt, Wittenberg, Wartenburg, NSG Großer Streng, 14-Oct. 2021, N 51.826944 E 12.773055, leg. Lars Hendrich”, “ZSM_HET_1402”, idem, “ZSM_HET_1403“, “ZSM_HET_1408–1411“, “ZSM_HET_1413”, “ZSM_HET_1414”, “ZSM_HET_1416”, “ZSM_HET_1421–1425”, “ZSM_HET_1428”, “ZSM_HET_1429”, “ZSM_HET_1431–1436”, “ZSM_HET_1441–1444”, “ZSM_HET_1449”, “ZSM_HET_1451”, “ZSM_HET_1455–1461”, “ZSM_HET_1464”, “ZSM_HET_1466”, “ZSM_HET_1470–1472”, “ZSM_HET_1474”, “ZSM_HET_1475”, “ZSM_HET_1477–1479”, “ZSM_HET_1481”, “ZSM_HET_1486”, “ZSM_HET_1487”, “ZSM_HET_1495–1497”, “ZSM_HET_1500”, “ZSM_HET_1501”.

**Greece:** 1 specim., “Greece, Corfu, Parelion, Ropa river east of Ermones, 07-Jun-2023, N 39.613611 E 19.793611, leg. unknown” “ZSM_HET_1503”.

**Switzerland:** 3 specim., “Switzerland, Tessin, Bolle di Magadino, 12-Jun-2017, N 46.148696 E 8.873741, leg. Rolf Niedringhaus”, “ZSM_HET_1008”, idem, “ZSM_HET_1009”, “ZSM_HET_1010”.

All 69 paratypes are provided with a red printed paratype label and deposited in the SNSB-ZSM.

### Additional material examined

Further material examined included 74 adult specimens (36 males and 38 females). Female specimens were only analyzed when their identity was confirmed verified by DNA barcodes.

**Austria:** 2 specim., “Austria, Burgenland, Neusiedl am See, Podersdorf am See, 23-May-1972, N 47.854722 E 16.829722, leg. E.-G. Burmeister”, “ZSM_HET_1381–1382”.

**Croatia:** 3 specim., “Croatia, Istria, Tinjan, 13-Aug-1979, N 45.216389 E 13.840278, leg. E.-G. Burmeister”, “ZSM_HET_1378–1380”.

**Germany:** 1 specim., ”Germany, Baden-Württemberg, Möggingen, Mindelsee, N 47.754444 E 9.022222, leg. Ralf Heckmann”, “ZSM_HET_1361“; 3 specim., “Germany, Lower Saxony, Ammerland, Jaderberg, 03-Jun-2016 N 53.334533 E 8.185283, leg. Nadine Lange”, “ZSM_HET_0819”, idem, “ZSM_HET_0824”, “ZSM_HET_0830” ; 9 specim., “Germany, Lower Saxony, Schwanewede, Harrier Sand, 14-Sep-2016, N 53.309271 E 8.499151, leg. Rolf Niedringhaus”, “ZSM_HET_0731”, idem, “ZSM_HET_0732 – ZSM_HET_0739”; 5 specim., “Germany, Baden-Württemberg, Ravensburg, Altshausen, Stuben, 29-Jul-2016, N 47.902321 E 9.575554, leg. Martin Gossner”, “ZSM_HET_0740“, idem, “ZSM_HET_0741“, “ZSM_HET_0742“, “ZSM_HET_0744“, “ZSM_HET_0745“; 6 specim., “Germany, Bavaria, Schwaben, Memmingen, Benninger Ried, 27-May-2017, N 47.975275 E 10.200967, leg. Martin Gossner”, “ZSM_HET_0803“, idem, “ZSM_HET_0804–0808”; 5 specim., “Germany, Brandenburg, Cottbus, 08-May-2016, N 51.760844 E 14.327172, leg. Rolf Niedringhaus”, “ZSM_HET_0746“, idem, “ZSM_HET_0747”, “ZSM_HET_0750”, “ZSM_HET_0762”, “ZSM_HET_0765”; 1 specim., “Germany, Brandenburg, Elbe-Elster Kreis, Stechau, Sandsee, 01-June-2020, N 51.684167 E 13.458056, leg. Lars Hendrich”, “ZSM_HET_1189“; 6 specim., “Germany, Brandenburg, Havelland, NE Götzer Berge, alder carr, 26-Aug-2020, N 52.4475 E 12.728611, leg. Lars Hendrich”, “ZSM_HET_1177“, idem, “ZSM_HET_1353–1357”; 9 specim., “Germany, Brandenburg, Prignitz, Lenzen an der Elbe, 10-Jul-2018, N 53.072039 E 11.479256, leg. Lars Hendrich”, “ZSM_HET_0939“, idem, “ZSM_HET_0940”, “ZSM_HET_0942”, “ZSM_HET_0944–0947”, “ZSM_HET_0949”, “ZSM_HET_0950”; 3 specim., “Germany, Lower Saxony, Ammerland, Jaderberg, 03-Jun-2016, N 53.334533 E 8.185283, leg. Rolf Niedringhaus”, “ZSM_HET_0751“, idem, “ZSM_HET_0752”, “ZSM_HET_0753”; 4 specim., “Germany, Lower Saxony, Ammerland, Jaderberg, 21-May-2017, N 53.335093 E 8.181291, leg. Nadine Lange”, “ZSM_HET_0821“, idem, “ZSM_HET_0825”, “ZSM_HET_0826”, “ZSM_HET_0829”; 7 specim., “Germany, Lower Saxony, Ammerland, Jaderberg, 25-May-2017, N 53.335093 E 8.181291, leg. Nadine Lange”, “ZSM_HET_0792“, idem, “ZSM_HET_0793–0795”, “ZSM_HET_0800–0802”; 6 specim., “Germany, Saarland St. Wendel, Nonnweiler, Otzenhausen, 04-Aug-2018, N 49.60425 E 6.994806, leg. Martin Gossner”, “ZSM_HET_0997“, idem, “ZSM_HET_0999”, “ZSM_HET_1001”, “ZSM_HET_1004”, “ZSM_HET_1006”, “ZSM_HET_1007”.

**Greece:** 4 specim., “Greece, Central Macedonia, Chalkidiki, Sithonia, Sarti, 27-Aug-2015, N 40.094581 E 23.976442. leg. Rolf Niedringhaus”, “ZSM_HET_0774”, idem, “ZSM_HET_0776”, “ZSM_HET_0992”, “ZSM_HET_0994”.

**Netherlands:** 4 specim., “Netherlands, Gelderland, Betuwe, Buren, 23-Sep-2015, N 51.908 E 5.319, leg. Ping-ping Chen, “ZSM_HET_0870”, idem, “ZSM_HET_0871”, “ZSM_HET_0908”, “ZSM_HET_0909”; 2 specim., “Netherlands, North Brabant, Oss, Herpen, 29-Sep-2015, N 51.760 E 5.611, leg. Ping-ping Chen”, “ZSM_HET_0857”, idem, “ZSM_HET_0900”.

**Romania:** 5 specim., “Romania, Siebenbürgen, Cluj, Cluj-Napoca, Visea, 14-May-2000, N 46.856389 E 23.874167, leg. M. Thiel”, “ZSM_HET_1371–1375”.

**Ukraine:** 3 specim., “Ukraine, Transcarpathia, Chust, Chust, 22-Apr-2018, N 48.164104 E 23.325518 leg. Martin Gossner”, “ZSM_HET_0931”, idem, “ZSM_HET_0932”, “ZSM_HET_0934”.

### Description

All measurements are given in mm.

### Body size

Body length 2.10–2.60, average 2.38 (holotype: 2.50). See Supplementary Movie S2 for a movie of a representative specimen.

### Color

Variable even among individuals within a population. Base body color almost white, to bright yellow over light brown to dark brown; with irregular honeycomb-like indentations with setae at the bases of the pits, especially on the pronotum; scutellum usually at bit lighter than wings and pronotum; legs yellowish-brown, distal and proximal ends usually darker (holotype: dark brown); venter usually darker than dorsum; eyes dark red to black; vertical light brown to dark brown bar between eyes (Fig. 4D–F).

### Head

Head generally concolorous with body, sometimes more intense; mouthparts usually dark brown; light brown to dark brown vertical bar along midline frontally, between eyes; bar from narrow, blurred light brown to broad, well-defined dark brown (well defined brown bar on holotype); antenna three-segmented, usually visible below eye; interocular distance from edge towards vertex 0.63–0.76 (holotype: 0.70), from edge towards mouthparts 0.50–0.56 (holotype: 0.53).

### Pronotum

Base color ranging from cream to light brown; irregular honeycombing apparent on most specimens; number, size, and shape of indentations highly variable; most punctures with setae at bases; holotype with some light brown regions at posterior and lateral edges.

### Wings

Complete to posterior end of body; punctures (0.02 in diameter) evenly distributed over entire hemelytra, without defined pattern and underlying irregular honeycomb structure; claval suture distinct, complete; scutellum as long as wide, base color amber-colored to light brown (holotype: amber); pits usually with dark base, especially on posterior region of wings; edge of corium usually darker than rest of wing; hindwing membranous, fully developed, completely concealed by hemelytra.

### Legs

Mesothoracic leg shortest; metathoracic leg longest; tibiae pilose, femora of all legs sparely covered with setae, often with single long seta near base; tarsal formula 3-3-3; average leg measurements: prothoracic coxa: 0.05, trochanter: 0.15, femur: 0.60, tibia: 0.70, tarsomere I: 0.03 mm, tarsomere II: 0.10, tarsomere III: 0.15, pretarsal claw: 0.10, total leg length: 1.88; mesothoracic coxa: 0.05, trochanter: 0.15, femur: 0.63, tibia: 0.50, tarsomere I: 0.03, tarsomere II: 0.13, tarsomere III: 0.15, pretarsal claw: 0.1, total leg length: 1.74; metathoracic coxa: 0.05, trochanter: 0.20, femur: 0.75, tibia: 0.70, tarsomere I: 0.05, tarsomere II: 0.25, tarsomere III: 0.28, pretarsal claw: 0.15, total leg length: 2.43.

### Median ventral keel

Anteriorly with an almost rectangular part beneath head, followed by angular prosternal keel with rounded edges and small posterior tooth; mesothoracic keel almost angular, inclined posteriorly; metathoracic keel irregularly shaped, with slot; first abdominal keel fused to metathoracic keel, followed by three almost fused parts with posterior teeth; four distinct teeth on first four abdominal segments (see *Plea m. minutissima*) (see Supplementary Fig. S1B).

### Female characters

Ovipositor roughly triangular, about 0.40 long; width 0.11 at widest point; posterior border (apical row) with 5–10; surface with 7–13 teeth (see Supplementary Fig. S2); subgenital plate subtriangular; average length 0.55; with slightly rugose surface on vaulted basal part, apically followed by series of pits with setae; surface darker on posterior third, sparsely covered with setae; median slot on posterior third.

### Male characters

Subgenital plate slightly longer than wide; average length 0.55 (holotype: 0.53); average width 0.49 (holotype: 0.48); without vaulted basal area as in females; surface slightly rugose, with short setae throughout; posterior third with slightly longer setae; right paramere curved at right angles; apex rounded; average length 0.19 (holotype: 0.19) left paramere more slender and elongated than in *Plea m. minutissima* (Fig. 5B); average length 0.2 (holotype: 0.2); stretched in an arch shape; base with folding.

### Habitat

See *Plea m. minutissima*. Eutrophic exposed and more permanent water bodies, rich in vegetation and with muddy bottom (Fig. 6D–F).

### Distribution

Albania, Austria, Croatia, Finland, Germany, Greece, Netherlands, Romania, Switzerland, and the Ukraine. This species can be expected in other eastern and south-eastern parts of Europe, e.g., Poland, the Czech Republic or Russia, Slovakia, and Hungary^14^, but has to be confirmed.

### Etymology

The specific epithet is based on the Latin adjective *crypticus* (cryptic) and refers to the long-overseen existence of this species.

### Key

1. Left male paramere compact, hook-shaped; apex distinctly rounded; base without folding (Fig. 5A). Distribution: Armenia, Belgium, France, Germany, Italy, Morocco, Spain, Tunisia, Turkey, and United Kingdom (England). *Plea minutissima minutissima*.

- Left male paramere more slender and elongated, stretched in an arch shape; base with folding (Fig. 5B). Distribution: Albania, Austria, Croatia, Finland, Germany, Greece, Netherlands, Romania, Switzerland, and the Ukraine *Plea cryptica* sp. nov.

## Discussion

Every year, about 17,000 species are newly described worldwide, primarily in tropical regions, with insects representing the largest fraction of these discoveries^53^. Notably, the process of formally describing and naming a new species typically takes an average of 21 years from its initial discovery^54^. While the discovery of a new insect species per se is not uncommon, it is a peculiarity for a well-studied group such as the European water bugs, which have been intensively studied for over two centuries. The recent identification of the overlooked *Plea* species was facilitated by the establishment of an extensive DNA barcode library for German water bugs^34^, highlighting the efficacy of molecular techniques in species identification^36^.

Despite convincing molecular data, morphological differences between the two *Plea* species are subtle, likely contributing to the oversight of *Plea cryptica* sp. nov. Several morphological features have historically served as useful characters to delineate species within the Pleidae. These include, for example, the state of body sculpturing, the widths of the pronotum and scutellum, the profile of the sternum, the male and female opercula, the male parameres, the form of the clavus^55^, or the tooth pattern of the female ovipositor^56^. In addition, Cook^3^ added body shape indices, an ocular index, a pronotal index, a scutellar index, and a scutellar length index to assess species in Pleidae. Unfortunately, all these characteristics are not useful to differentiate the two *Plea* species treated here, as they exhibit a high degree of variability within both species. Consequently, a reliable morphological species determination is feasible only in males, based on the shape of the left paramere (Fig. 5). This situation is akin to other water bugs, for example within the genera *Anisops*^57,58^, *Buenoa*^58^, or *Sigara*^59^. Conversely, a valid identification of females relies solely on sequence data.

In this context we emphasize that *Plea cryptica* sp. nov. does not correspond to the subspecies *Plea m. tassilii*. This subspecies was described by Poisson in 1953 as *forma*^13^ and upgraded by Stichel^47^. So far, it is only known from the isolated Tassili n’Ajjer plateau in southeastern Algeria at the borders of Libya, Niger, and Mali in the Sahara Desert^13^. A careful comparison of the right parameres shows that the strongly curved part of *Plea cryptica* sp. nov. is much more strongly curved and elongated compared to *Plea m. tasslilii*^13^. In addition, a significantly narrower distal part of the paramere of *Plea cryptica* sp.nov. is separated from the thicker middle part, while for *Plea m. tassilii* the middle and distal parts are even merge. Interestingly, the drawing of the parameres of *Plea leachi* = *Plea m. minutissima* in Poisson`s work^13^ is somewhat peculiar and does not correspond to other drawings as it is shown some years later in his Fauna de France volume^60^. It is therefore entirely possible that the figure legends were not assigned correctly. In our opinion *Plea minutissima tassilii* represents an endemic subspecies or even distinct species.

Differences in the life history and ecological niches (Fig. 6) between the two *Plea* species remain unexplored, although it can be assumed that they probably do not differ significantly as this would probably have been noticed in the past. Appropriate studies should be conducted in the future. Similarly, differences in bioacoustics may provide additional insights into species behavior and communication^31^. However, the median ventral keels of both species are very similar (Supplementary Fig. S1).

**Figure 7:**
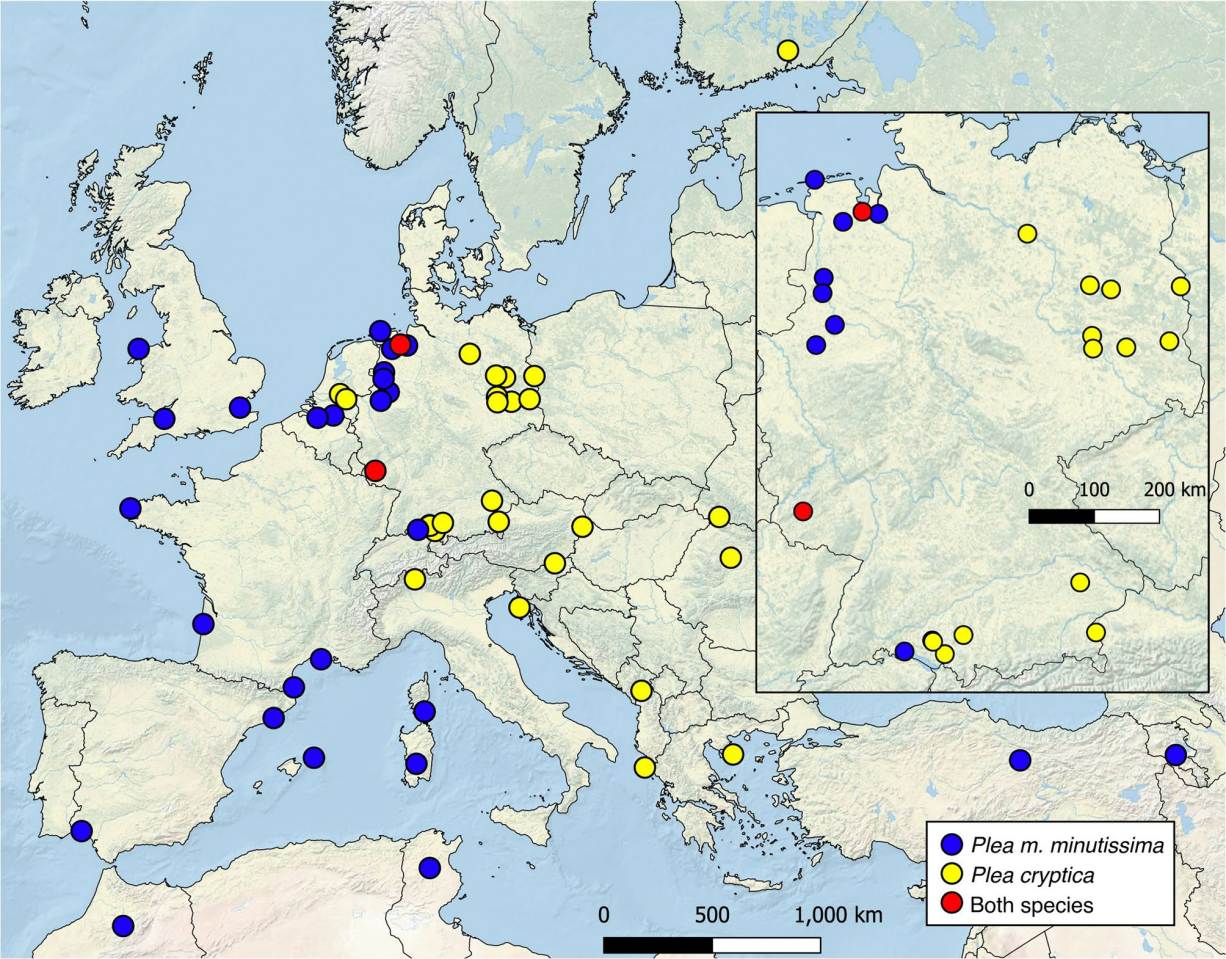
Geographic distribution of *Plea m. minutissima* Leach, 1817 (blue), *Plea cryptica* sp. nov. (yellow), and both species (red) based on the studied samples. The map was created with QGis 3.36.2 (www.qgis.org/de/site/index.html).

Our data suggest that *Plea m. minutissima* is mainly distributed in Western Europe, Northern Africa, and the Near East to Armenia, whereas *Plea cryptica* sp. nov. is a more common in Central and Eastern Europe, with documented sympatric occurrences at least in regions in Lower Saxony and the Saarland in Germany (Fig. 7; Table 4). However, defining precise boundary lines between their distributions remains challenging, suggesting the likelihood of additional overlap areas. Since the data known on the distribution of *Plea m. minutissima* include various countries that we were unable to analyze in our study, we assume that *Plea cryptica* sp. nov. will replace *Plea m. minutissima* in other countries, such as Poland, the Czech Republic, Slovakia, Hungary, Serbia or Belarus. Nevertheless, this is outside the scope of this work and has to be done by local scientists.

**Table 4:** Summary of the sample localities of all analyzed specimens of *Plea m. minutissima* Leach, 1817 and *Plea cryptica* sp. nov.

Country	Federal State	Sector	Collection Site	*Plea m. minutissima* DNA Barcodes	*Plea m. minutissima* Morphology	*Plea cryptica* sp. nov. DNA Barcodes	*Plea cryptica* sp. nov. Morphology
Albania	Shkodra	Ducaj				3*	
Armenia	Tavush	Zovk			1		
Austria	Burgenland		Podersd. am See				2
Austria	Styria	Leutschach				2	
Belgium	Antwerpen	Turnhout	Tikkebroeken	3			
Belgium	East Flanders	Beveren		21			
Croatia	Istria		Tinjan				3
Finland	Kymenlaakso	Kouvola				3*	
France	Bretagne	Finistre	Saint-Renan		3		
France	Corse	Lecci	Cala Rossa	3			
France	Corse	Lecci	River L’ Oso	7			
France	Gironde	Arcachon	La Teste		2		
France	PACA	Bouches-du-Rh.	St.-Mar.-de-la-M.		3		
France	Pyrénées Orientales		Banyuls-sur-Mer		2		
Germany	Baden-Württemb.	Möggingen	Mindelsee		2		
Germany	Baden-Württemb.	Wangen	Großweiher			3	
Germany	Baden-Württemb.	Altshausen				6	
Germany	Baden-Württemb.	Wolpertswende	Blindsee			3	
Germany	Bavaria	Thürnthenning	Königsauer Moos			2 / 3*	
Germany	Bavaria	Memmingen	Benninger Ried			6	
Germany	Bavaria	Fridolfing	Haslau			1*	
Germany	Berlin	Wannsee	Teltowkanal			13	
Germany	Brandenburg	Cottbus				4 / 3*	4
Germany	Brandenburg	Stechau				1	
Germany	Brandenburg	Götzerberge					5
Germany	Brandenburg	Lebus				5	
Germany	Brandenburg	Lenzen (Elbe)				10	
Germany	Lower Saxony	Jaderberg		6 / 1*		17 / 2*	
Germany	Lower Saxony	Apen	Aper Tief	2			
Germany	Lower Saxony	Lingen	Brockhausen	7 / 4*			
Germany	Lower Saxony			10 / 3*			
Germany	Lower Saxony	Schwanewede	Harriersand	9			
Germany	North Rhine-West.	Haltern	Quarzwerke		4		
Germany	North Rhine-West.	Münster			1		
Germany	North Rhine-West.	Recke	Heiliges Meer	2	1		
Germany	Saarland	Nonnweiler	Otzenhausen	2		6	
Germany	Sachsen-Anh.	Wartenburg	Großer Streng			1	53
Germany	Sachsen	Bad Schmiedeb.				4	
Greece	Central Macedonia	Sithonia	Sarti			8	
Greece	Korfu						1
Italy	Sardinia	Oristano	Giara di Gesturi		2		
Morocco	Ifrane	Fez			1		
Netherlands	Gelderland	Betuwe	Buren			4	4
Netherlands	North Brabant	Oss	Herpen				2
Romania	Siebenbürgen	Cluj	Visea				5
Spain	Andalusia	Huelva			5		
Spain	Balearic Islands				4		
Spain	Catalunya	Barcelona			1		
Switzerland	Tessin	Bolle di Magadi.	Switzerland				3
Tunisia	Kairouan	Oueslatia			1		
Turkey	Sivas	Demiryurt	Tödürge Gölü		1		
Ukraine	Transcarpathia	Chust				4	
Unit. Kingdom	Anglesey	Llanddaniel		1*			
Unit. Kingdom	Essex	Canvey Island		2			
Unit. Kingdom	Somerset	Somerset Lev.	Pawlett Hams	5	1		
**Total**				**88**	**35**	**114**	**82**

Studied specimens are separated as to whether they were examined molecularly (DNA barcodes) or strict morphologically. Asterisks indicate published DNA barcodes and their sampling locality (see Material and Methods).

The discovery of a hitherto unknown water bug species in Europe shows that new species can be discovered even in well-studied regions. It also highlights the usefulness of DNA barcoding in modern taxonomic research. For example, the analysis of more than 48,000 DNA barcodes from Diptera collected via Malaise traps deployed in southern Germany revealed evidence of at least 1,800 to 2,200 unknown species within the Cecidomyiidae, Chironomidae, Phoridae, and Sciaridae^61^. Consequently, the availability of a diverse range of molecular biological, morphological, and cybertaxonomic methods has greatly enhanced the ability to conduct detailed species descriptions^36^. As a result, taxonomic research has become more attractive and robust than ever before, offering unprecedented opportunities for exploration and discovery in the field of biodiversity science.

## Materials and methods

### Specimens analyzed

Specimens were collected in different locations across Europe during the last few years (see Supplementary Table S3), using hand nets or sieves, and fixed in ethanol (96%). In addition, various mounted specimens were examined. When individuals were analyzed morphologically, they were mounted after the examination. Female specimens were only analyzed morphologically after their identities were verified by DNA barcodes. In total, 300 specimens were analyzed. Detailed information about which specimens were examined by morphology (*P. m. minutissima*: *n* = 34; *P. cryptica* sp. nov: *n* = 88), molecular data (42; 35), or both approaches (37; 64), can be found in the Supplementary Table S3. All specimens analyzed, except for the neotype of *P. m. minutissima*, were deposited at the Bavarian State Collection of Zoology (SNSB-ZSM), Germany.

### Molecular studies

#### DNA isolation

We selected 178 specimens exhibiting morphological characteristics consistent with *P. m. minutissima* for molecular analyses. Total genomic DNA was extracted from 2 to 3 dissected legs or, in rare cases, from complete specimens, using the NucleoSpin Tissue Kit (Macherey-Nagel), following the manufacturer`s extraction protocol. These procedures were conducted at the Bavarian State Collection of Zoology (SNSB-ZSM), Germany (Voucher codes: see Supplementary Table S3).

#### DNA barcoding: amplification, sequencing, and analyses

Barcode amplicons were amplified using illustraTM puReTaq Ready-To-Go PCR Beads (GE Healthcare, Buckinghamshire, UK) in a total volume of 20 µl. The reaction mixture comprised 17.5 µl sterile molecular grade H_2_O, 2 µl DNA template with a concentration between 2 and 150 ng/µl and 0.25 µl of each primer (20 pmol/µl, primer pair LCO1480 and HCO1480)^62^. The thermal cycling protocol consisted of an initial denaturation at 94 °C for 5 min, followed by 38 cycles of denaturation at 94 °C for 45 s, annealing at 48 °C for 45 s, extension at 72 °C for 80 s, and a final extension step at 72 °C for 7 min. All PCR amplification reactions were conducted using an Eppendorf Mastercycler Pro system (Eppendorf, Hamburg, Germany). Three µl of the amplified products were subjected to size verification by electrophoresis in a 1% agarose gel stained with GelRed™ using commercial DNA size standards. The verified PCR products were purified with the NucleoSpin Gel and PCR Clean-up Kit (Macherey-Nagel, Düren, Germany). Purified PCR products underwent cycle sequencing in both directions at contract sequencing facilities (Macrogen, Seoul, Korea, or GATC, Konstanz, Germany), with the same primers used in PCR. Double stranded sequences were assembled and checked for mitochondrial pseudogenes (numts) by analyzing the presence of stop codons, frameshifts, and double peaks in chromatograms using Geneious Prime 2022.0.1 (https://www.geneious.com) (Biomatters, Auckland, New Zealand). For validation purposes, BLAST searches (nBLAST, search set: others, program selection: megablast) were conducted to confirm the identity of all new sequences as pygmy backswimmer barcodes, based on already published sequences, with high identity values and very low E-values.

The DNA barcode dataset was analyzed following an established workflow, consistent with methodologies employed in previous studies^34,63^. Analysis tools provided by the Barcode of Life Data System workbench (BOLD; www.boldsystems.org)^64^ were utilized for this purpose. To enhance the dataset, we incorporated all publicly available DNA barcodes of *P. m. minutissima* from BOLD (*n*= 24) with 3 specimens from Albania^65^, 1 specimen from England (no publication), 3 specimens from Finland^66^, and 17 specimens from Germany^34,63^. The reference date for accessing this data was December 3rd, 2023.

In addition to *P. m. minutissima*, we expanded the dataset to include all available DNA barcodes with at least 400 bp and a taxonomic classification at the species level for other Pleidae. This encompassed the species *Neoplea striola* (*n*= 3)^34^, *Paraplea brunni* (Kirkaldy, 1898)^67^ (*n* = 2), *Paraplea frontalis* (Fieber, 1844)^35^ (*n* = 1), *Paraplea halei* (Lundblad, 1933)^68^ (*n* = 1), *Paraplea indistinguenda* (Matsumura, 1905)^69^ (*n* = 1), and *Paraplea liturata* (Fieber, 1844)^35^ (*n* = 1). Additionally, 10 DNA barcodes from the backswimmer *Notonecta glauca* Linnaeus, 1758^23^ were included as outgroup taxa^33^. Consequently, the complete dataset comprised a total of 221 DNA barcodes.

We used the BOLD workbench to calculate the nucleotide composition of the sequences and distributions of Kimura-2-parameter distances (K2P)^70^ within and between species (align sequences: BOLD aligner; ambiguous base/gap handling: pairwise deletion). All barcode sequences became subject of the Barcode Index Number (BIN) analysis system implemented in BOLD that clusters DNA barcodes to produce operational taxonomic units that typically closely correspond to species^71^. Initially, a threshold of 2.2% was applied to roughly differentiate between intra- and interspecific distances. Subsequently, further refinements through Markov clustering, resulting in the assignment of sequences into final BINs. These BIN assignments on BOLD can change and are constantly updated as new sequences are added, leading to splitting and/or merging of individual BINs in the light of new data^41^.

All sequences were aligned using MAFFT 7.45 based on the FFT-NS-2 algorithm^72,73^ and analyzed using a neighbor-joining cluster analysis (NJ)^74^ based on K2P distances with MEGA 11^75^. Non-parametric bootstrap support values were obtained by resampling and analyzing 1,000 replicates^76^. It should be noted that this analysis is not intended to be phylogenetic inference; rather, the resulting topology represents a graphical visualization of DNA barcode distance divergences and species clustering. Comprehensive voucher information, taxonomic classifications, photos, DNA barcode sequences, primer pairs and trace files are publicly accessible through the public dataset “DS-SDGP” on the Barcode of Life Data Systems (BOLD; www.boldsystems.org)^64^.

### Genome skimming: mitochondrial genomes and nuclear rRNA genes

High-throughput sequencing technology was applied to obtain mitochondrial genome sequences of one representative of both *Plea* species and one specimen of *N. striola*. The sequence libraries were constructed using the NEBNext Ultra FS DNA Library Prep Kit at a commercial sequencing facility (StarSEQ GmbH, Mainz, Germany), followed by sequencing on an Illumina NextSeq 500 with a 150-base paired-end approach and an estimated output of 10 million reads per sample. Quality control of demultiplexed reads was performed using FastQC (Version 0.11.9) (https://www.bioinformatics.babraham.ac.uk/projects/fastqc/*)*, while removal of Illumina adapters was carried out using cutadapt (v3.1)^77^. Contigs assembly was done using SPAdes (v3.13.0)^78^.

Identification of contigs potentially representing mitochondrial genomes was based on length, GC-content, and coverage. In a subsequent step, all reads were remapped onto the putative mitochondrial genomes to reanalyze low-coverage regions, gaps and misassembles. Final editing and annotation were performed with Geneious Prime 2022.0.2 (https://www.geneious.com*)*, by using the mitochondrial genome of the *Paraplea frontalis*as matrix^79^ and results of MITOS analysis^80^.

Following the concept of concerted evolution of nuclear rRNA genes^81–83^, even single base substitutions within the hypervariable expansion segments can be used for the identification of closely related but distinct species^84^. Complete 18S rRNA and 28S rRNA gene sequences were mined from the three sequence libraries using the following gene sequences as baits: 18S rRNA gene: *Lygus hesperus* Knight, 1917^85^ (accession number: U06476)^86^; 28S rRNA gene: *Aquarius paludum* (Fabricius, 1794)^87^(KX821839)^88^.

### Complete nuclear 18S: amplification, sequencing, and analyses

Additional complete nuclear 18S rRNA genes were amplified via polymerase chain reaction (PCR) for two selected individuals of each *Plea*species. Detailed information about used primer pairs, amplification reactions and temperature profiles for the amplification and sequencing of the 18S rRNA genes are provided in previous studies^89,90^. For verification, negative and positive controls were included in each round of reactions. Three µl of the amplified products were inspected for size conformity by electrophoresis in a 1.5% agarose gel with GelRed™ using commercial DNA size standards. The remaining PCR product was purified with the QIAquick PCR Purification Kit (Qiagen GmbH, Hilden, Germany). Purified PCR products were cycle sequenced and sequenced in both directions at a contract sequencing facility (GATC, Konstanz, Germany) using the same primers as employed in the PCR as well as some internal primers^91^. Complementary sequences were assembled with Geneious Prime 2022.0.1. Finally, BLAST searches were performed to confirm the identity of all new 18S and 28S rRNA gene sequences.

Complete sequences were aligned using MAFFT 7.45 based on the FFT-NS-2 algorithm. For comparison, two rRNA gene sequences of *Paraplea frontalis* were added (18S: KJ461252^92^; 28S: KJ461196^92^. Patristic distances (p-distances) of all sequences were calculated using MEGA 11. New sequences are publicly accessible through the public dataset “DS-SDGP” on the Barcode of Life Data Systems and were deposited in GenBank (see Supplementary Table S3).

### Mitochondrial genomes: sequence alignment and phylogenetic analyses

In addition to the three new mitochondrial genomes, all published mitogenomes of the Notonectoidea available on August 15th, 2023, were included in the analyses (see Supplementary Table S4). Furthermore, two mitochondrial genomes of the Saldidae (Leptopodomorpha) were retrieved from GenBank and selected as outgroup. Thus, the combined data set included 13 species.

All protein-coding and both rRNA genes were aligned individually using the Q-INS-i algorithm as implemented in MAFFT 7.45 with default settings. Manual inspection of the alignments was performed in Geneious Prime 2022.0.2. To remove poorly aligned positions and divergent regions of the 12S and 16S rRNA gene alignments, Gblocks 0.91b^93,94^ was applied with the less stringent selection option (allow smaller final blocks, allow gap positions within the final blocks, allow less strict flanking positions). Consequently, the 12S rRNA gene alignment was reduced from 816 base pairs (bp) to 750 bp (92% of the original alignment length) and the 16S rRNA gene alignment from 1,292 bp to 1,220 bp (94%). Subsequently, all genes or gene fragments (i.e., rRNAs) were concatenated, resulting in an alignment with a total length of 13,172 bp.

Phylogenetic relationships among the selected taxa were inferred under the maximum likelihood (ML) criterion using IQ-TREE 2.1.2^95^. The best model of nucleotide substitution was determined for each gene based on the Bayesian Information Criterion (BIC) with Modelfinder^96^. A summary of all models used in the dataset is provided in the supporting information (Supplementary Table S5). To assess nodal support, 10,000 ultrafast bootstrap replicates^97^ and 10,000 replicates of an SH-aLRT test^98^ were performed. Ultrafast bootstrapping (UFBoot) has been demonstrated to be largely unbiased compared to standard or alternative bootstrapping, whereas SH-aLRT values are as conservative as standard non-parametric bootstrap values^99^. Typically, nodes with support values of UFBoot ≥ 95% and SH-aLRT ≥ 90% were considered as very robust and values ≥ 80% as robust^97,99^.

### Morphological studies

Observations and measurements were made using a Leica M 205 C stereomicroscope equipped with a photo tube and ocular micrometer. Selected specimens were photographed using a Panasonic Lumix G9 MFT camera, along with a Novoflex adjusting slide Castel-Micro and a Novoflex MICRO-TUBE special tube, in combination with Mitutoyo objectives (10X and 20X). We follow diagnoses, terms, and definitions of previous taxonomic studies on Pleidae^3,6–8^.

### 3D scanning and modelling

Image acquisition was done using the DISC3D*plus*prototype^100^ developed by Small World Vision GmbH (Darmstadt, Germany). A total of 392 extended-depth-of-field images were obtained for each specimen, with each image composed of 43 stack images. The resulting images had a final resolution of 1876 × 1965 pixel for *P. m. minutissima* and 2201 × 2253 pixel for *P. cryptica* sp. nov., with a pixel pitch of 2.5 μm at a magnification of 1.35. The 3D models creation (i.e., photogrammetry) was performed using Metashape Professional 2.1.1 (Agisoft LLC, St. Petersburg, Russia). Subsequently, the 3D model of *P. m. minutissima* was edited in Blender 4.1 (Blender Foundation, Amsterdam, Netherlands) to remove two artefacts (a hole in each eye). Post-processing of the texture was done with Adobe Photoshop 25.6.0 (Adobe Systems Software Ireland Limited, Dublin, Ireland). All renderings were done in Blender 4.1. Both movies were composed using Adobe Premiere Pro 24.3.0 (Adobe Systems Software Ireland Limited, Dublin, Ireland).

## Supplementary Information

Supplementary Material 1

Supplementary Material 2

Supplementary Material 3

Supplementary Material 4

Supplementary Material 5

Supplementary Material 6

Supplementary Material 7

Supplementary Material 8

Supplementary Material 9

Supplementary Material 10

## Data Availability

All new sequences (CO1, 18S, 28S) are publicly accessible through the public dataset “DS-SDGP” on the Barcode of Life Data Systems (BOLD; www.boldsystems.org) and were deposited in GenBank (see Supplementary Table S3 for accession numbers). New mitochondrial genomes were deposited in GenBank (accession numbers: *Neoplea striola*: PP405077, *Plea m. minutissima*: PP405075, *Plea cryptica* sp. nov.: PP405076). All data necessary to evaluate the conclusions of the paper are included in the paper and its supplementary information. The publication was registered in ZooBank: urn:lsid:zoobank.org:pub:BD490C7A-FACB-479E-9CBD-8F2B21CB134E.
