# Supplementary material for: The discovery of an overseen pygmy backswimmer in Europe (Heteroptera, Nepomorpha, Pleidae)
Source: Sci Rep. 2024 Nov 15;14:28139. doi: 10.1038/s41598-024-78224-6 (PMC11568165; doi:10.1038/s41598-024-78224-6)
Supplement: Supplementary file 1 — Supplementary Material 1 [file 41598_2024_78224_MOESM1_ESM.docx]

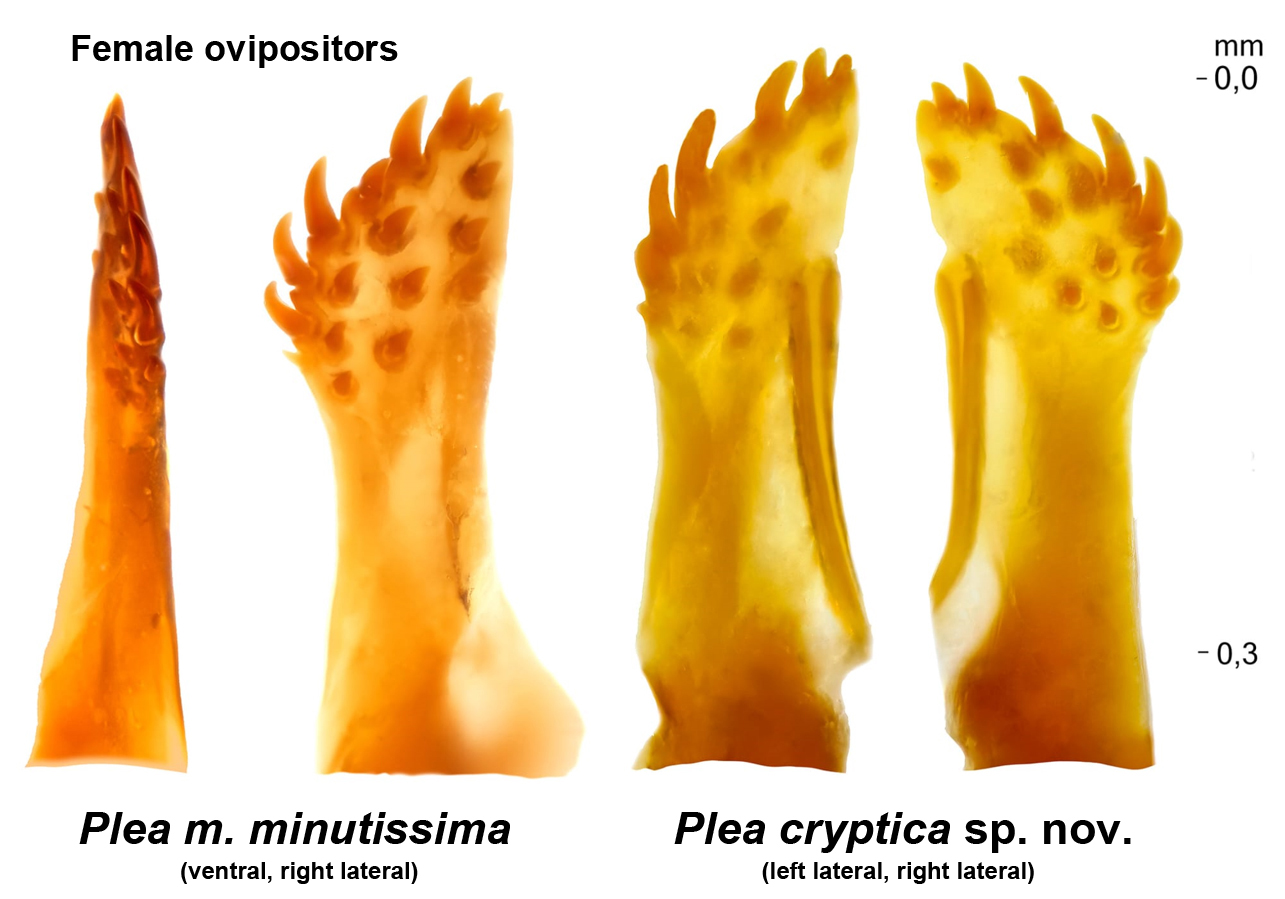


**Supplementary Figure S2:** Female ovipositors of *Plea m. minutissima* Leach, 1817 (ventral and right lateral), and *Plea cryptica* sp. nov (right and left lateral).
