# Supplementary material for: The discovery of an overseen pygmy backswimmer in Europe (Heteroptera, Nepomorpha, Pleidae)
Source: Sci Rep. 2024 Nov 15;14:28139. doi: 10.1038/s41598-024-78224-6 (PMC11568165; doi:10.1038/s41598-024-78224-6)
Supplement: Supplementary file 2 — Supplementary Material 2 [file 41598_2024_78224_MOESM2_ESM.docx]

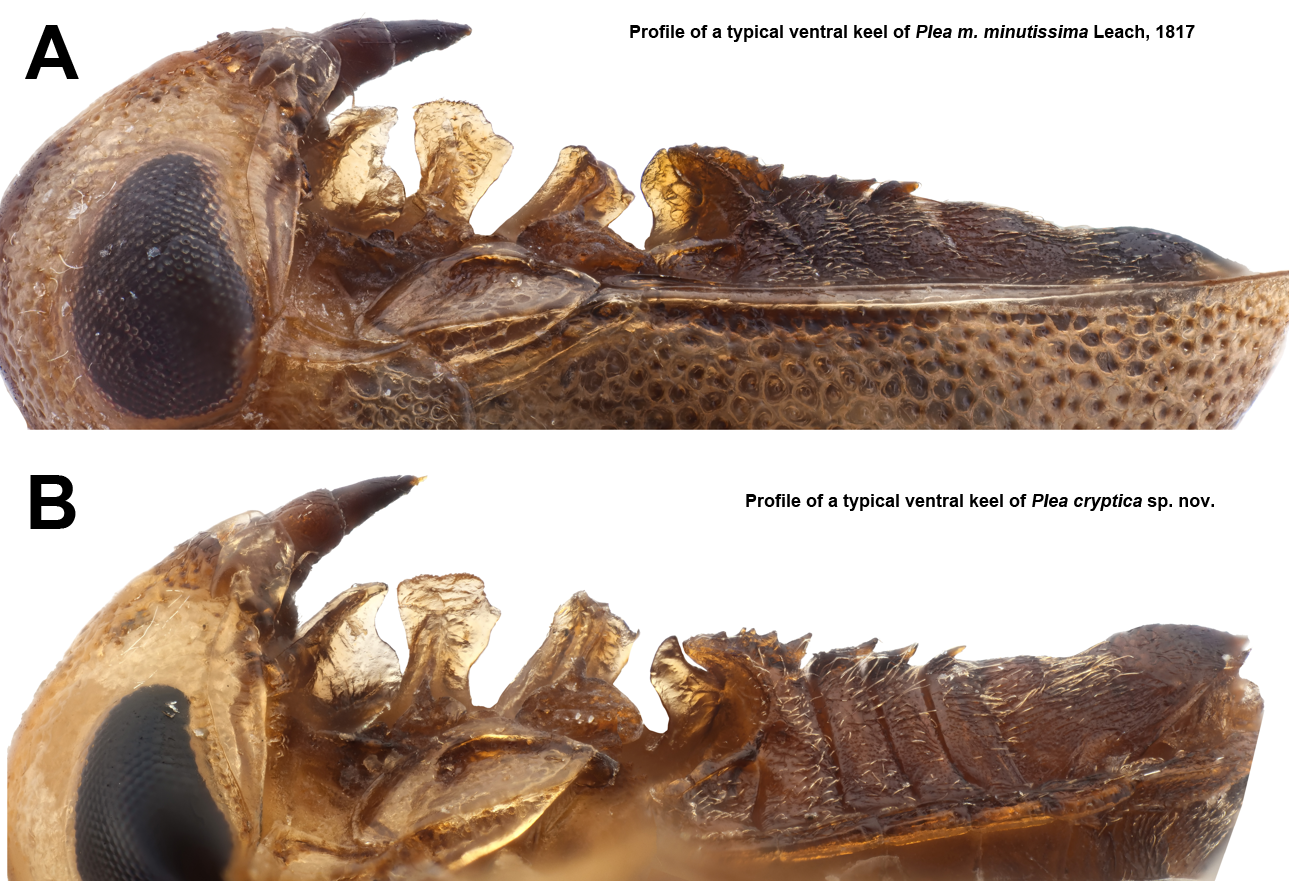


**Supplementary Figure S1:** Profile of a typical ventral keel of (A) *Plea m. minutissima* Leach, 1817 and (B) *Plea cryptica* sp. nov., with the anterior (thoracic) end to the left and the abdominal part to the right.
