# Supplementary material for: The discovery of an overseen pygmy backswimmer in Europe (Heteroptera, Nepomorpha, Pleidae)
Source: Sci Rep. 2024 Nov 15;14:28139. doi: 10.1038/s41598-024-78224-6 (PMC11568165; doi:10.1038/s41598-024-78224-6)
Supplement: Supplementary file 3 — Supplementary Material 3 [file 41598_2024_78224_MOESM3_ESM.docx]

**Supplementary Table S1:** Gene structures and arrangements of the mitochondrial genomes of *Plea m. minutissima* Leach, 1817 and *Plea cryptica* sp. nov. The letters H (heavy) or L (light) indicate that the gene is encoded either by the H- or L-strand. Numbers correspond to the nucleotides separating adjacent genes. Negative numbers indicate overlapping nucleotides.

| **Gene** | **Strand** | ***Plea m. minutissima*** | | | | | ***Plea cryptica* sp. nov.** | | | | |  |
| --- | --- | --- | --- | --- | --- | --- | --- | --- | --- | --- | --- | --- |
|  |  | **Start** | **Stop** | **Length (bp)** | **Amino acids** | **Intergenic** | **Start** | **Stop** | **Length (bp)** | **Amino acids** | **Intergenic** | ***p*-distance** |
| tRNA^Ile^ | H | 1 | 66 | 66 |  | -3 | 1 | 66 | 66 |  | -3 |  |
| tRNA^Gln^ | L | 64 | 132 | 69 |  | -1 | 64 | 133 | 70 |  | -1 |  |
| tRNA^Met^ | H | 132 | 201 | 70 |  | 0 | 133 | 202 | 70 |  | 0 |  |
| NAD2 | H | 202 | 1204 | 1003 | 334* | 0 | 203 | 1205 | 1003 | 334* | 0 | 0.105 |
| tRNA^Trp^ | H | 1205 | 1270 | 66 |  | -8 | 1206 | 1271 | 66 |  | -8 |  |
| tRNA^Cys^ | L | 1263 | 1327 | 65 |  | 0 | 1264 | 1329 | 66 |  | 0 |  |
| tRNA^Tyr^ | L | 1328 | 1395 | 68 |  | 1 | 1330 | 1397 | 68 |  | 1 |  |
| COI | H | 1397 | 2930 | 1534 | 511* | 0 | 1399 | 2932 | 1534 | 511* | 0 | 0.082 |
| tRNA^Leu2^ | H | 2931 | 2995 | 65 |  | 0 | 2933 | 2997 | 65 |  | 0 |  |
| COII | H | 2996 | 3674 | 679 | 226* | 0 | 2998 | 3676 | 679 | 226* | 0 | 0.075 |
| tRNA^Lys^ | H | 3675 | 3747 | 73 |  | 0 | 3677 | 3749 | 73 |  | -1 |  |
| tRNA^Asp^ | H | 3748 | 3814 | 67 |  | 0 | 3749 | 3814 | 66 |  | 0 |  |
| ATP8 | H | 3815 | 3970 | 156 | 52 | -7 | 3815 | 3970 | 156 | 52 | -7 | 0.109 |
| ATP6 | H | 3964 | 4641 | 678 | 226 | -11 | 3964 | 4641 | 678 | 226 | -11 | 0.077 |
| COIII | H | 4631 | 5417 | 787 | 262* | 0 | 4631 | 5417 | 787 | 262* | 0 | 0.079 |
| tRNA^Gly^ | H | 5418 | 5479 | 62 |  | 0 | 5418 | 5479 | 62 |  | 0 |  |
| NAD3 | H | 5480 | 5833 | 354 | 118 | 2 | 5480 | 5833 | 354 | 118 | 2 | 0.105 |
| tRNA^Ala^ | H | 5836 | 5901 | 66 |  | -1 | 5836 | 5901 | 66 |  | -1 |  |
| tRNA^Arg^ | H | 5901 | 5969 | 69 |  | -1 | 5901 | 5971 | 71 |  | -1 |  |
| tRNA^Asn^ | H | 5969 | 6039 | 71 |  | -1 | 5971 | 6036 | 66 |  | -1 |  |
| tRNA^Ser1^ | H | 6039 | 6110 | 72 |  | -1 | 6036 | 6107 | 72 |  | -1 |  |
| tRNA^Glu^ | H | 6110 | 6174 | 65 |  | -2 | 6107 | 6171 | 65 |  | -2 |  |
| tRNA^Phe^ | L | 6173 | 6239 | 67 |  | 0 | 6170 | 6238 | 69 |  | 0 |  |
| NAD5 | L | 6240 | 7944 | 1705 | 568* | 1 | 6239 | 7943 | 1705 | 568* | 1 | 0.094 |
| tRNA^His^ | L | 7946 | 8007 | 62 |  | 0 | 7945 | 8007 | 63 |  | 0 |  |
| NAD4 | L | 8008 | 9343 | 1336 | 445* | 0 | 8008 | 9343 | 1336 | 445* | 0 | 0.080 |
| NAD4L | L | 9343 | 9648 | 306 | 102 | -1 | 9343 | 9648 | 306 | 102 | -1 | 0.075 |
| tRNA^Thr^ | H | 9651 | 9715 | 65 |  | 2 | 9651 | 9719 | 69 |  | 2 |  |
| tRNA^Pro^ | L | 9716 | 9782 | 67 |  | 0 | 9720 | 9787 | 68 |  | 0 |  |
| NAD6 | H | 9785 | 10285 | 501 | 167 | 2 | 9790 | 10290 | 501 | 167 | 2 | 0.086 |
| COB | H | 10285 | 11419 | 1135 | 378* | -1 | 10290 | 11424 | 1135 | 378* | -1 | 0.084 |
| tRNA^Ser2^ | H | 11420 | 11487 | 68 |  | 0 | 11425 | 11492 | 68 |  | 0 |  |
| NAD1 | L | 11505 | 12431 | 927 | 309 | 17 | 11510 | 12436 | 927 | 309 | 17 | 0.067 |
| tRNA^Leu1^ | L | 12432 | 12495 | 64 |  | 0 | 12437 | 12500 | 64 |  | 0 |  |
| 16S rRNA | L | 12496 | 13764 | 1269 |  | 0 | 12501 | 13772 | 1272 |  | 0 | 0.055 |
| tRNA^Val^ | L | 13765 | 12835 | 71 |  | 0 | 13773 | 13843 | 71 |  | 0 |  |
| 12S rRNA | L | 13836 | 14612 | 777 |  | 0 | 13844 | 14629 | 786 |  | 0 | 0.045 |
| ORF | H | 14613 | 15338 | 726 |  | 0 | 14630 | 15624 | 995 |  | 0 |  |

Asterisks (*) indicate proteins with incomplete stop codons.
