# Supplementary material for: The discovery of an overseen pygmy backswimmer in Europe (Heteroptera, Nepomorpha, Pleidae)
Source: Sci Rep. 2024 Nov 15;14:28139. doi: 10.1038/s41598-024-78224-6 (PMC11568165; doi:10.1038/s41598-024-78224-6)
Supplement: Supplementary file 5 — Supplementary Material 5 [file 41598_2024_78224_MOESM5_ESM.docx]

**Supplementary Table S4:** Table containing all published mitochondrial genomes that were used in the phylogenomic analysis of the Notonectoidea.

| **Mitochondrial genomes obtained from NCBI** | **Infraorder** | **Family** | **Accession number** | **Reference** |
| --- | --- | --- | --- | --- |
| *Saldula arsenjevi* Vinokurow, 1981 | Leptopodomorpha | Saldidae | EU427345 | Hua et al. 2009 |
| *Saldula burmanica* Lindskog, 1975 | Leptopodomorpha | Saldidae | KY069963 | Jiang 2017 |
| *Helotrephes* sp. | Nepomorpha | Helotrephidae | NC_012822 | Hua et al. 2009 |
| *Helotrephes semiglobosus* Stål, 1860 | Nepomorpha | Helotrephidae | KJ027513 | No publication |
| *Enithares tibialis* Liu & Zheng, 1991 | Nepomorpha | Notonectidae | FJ456949 | Hua et al. 2009 |
| *Notonecta amplifica* Kiritshenko, 1931 | Nepomorpha | Notonectidae | MZ305077 | No publication |
| *Notonecta chinensis* Fallou, 1887 | Nepomorpha | Notonectidae | KX034036 | No publication |
| *Notonecta montandoni* Kirkaldy, 1897 | Nepomorpha | Notonectidae | MH780861 | No publication |
| *Notonecta triguttata* Motschulsky, 1861 | Nepomorpha | Notonectidae | FJ456951 | No publication |
| *Paraplea frontalis* (Fieber, 1844) | Nepomorpha | Pleidae | NC_208629 | Li et al. 2014 |

**References**

Hua J, Li M, Dong P, Cui Y, Xie Q, Bu W (2009) Phylogenetic analysis of the true water bugs (Insecta: Hemiptera: Heteroptera: Nepomorpha): evidence from mitochondrial genomes. BMC Ecology and Evolution 9: 134.

Jiang P. (2017) Studies on the comparative mitochondrial genomics and phylogeny of Heteroptera (Insecta: Hemiptera). PhD Thesis, China Agricultural University, Yuanmingyuan West Road, Beijing, Beijing 100193, China.

Li T, Hua J, Wright AM, Cui Y, Xie Q, Bu W, Hillis DM (2014) Long-branch attraction and the phylogeny of true water bugs (Hemiptera: Nepomorpha) as estimated from mitochondrial genomes. BMC Evolutionary Biology 14: 99.
