# Supplementary material for: The discovery of an overseen pygmy backswimmer in Europe (Heteroptera, Nepomorpha, Pleidae)
Source: Sci Rep. 2024 Nov 15;14:28139. doi: 10.1038/s41598-024-78224-6 (PMC11568165; doi:10.1038/s41598-024-78224-6)
Supplement: Supplementary file 6 — Supplementary Material 6 [file 41598_2024_78224_MOESM6_ESM.docx]

**Supplementary Table S2:** Gene structures and arrangements of the mitochondrial genomes of *Neoplea striola* (Fieber, 1844). The letters H (heavy) or L (light) indicate that the gene is encoded either by the H- or L-strand. Numbers correspond to the nucleotides separating adjacent genes. Negative numbers indicate overlapping nucleotides.

| **Gene** | **Strand** | ***Neoplea striola*** | | | | |
| --- | --- | --- | --- | --- | --- | --- |
|  |  | **Start** | **Stop** | **Length (bp)** | **Amino acids** | **Intergenic** |
| tRNA^Ile^ | H | 1 | 64 | 64 |  | -3 |
| tRNA^Gln^ | L | 62 | 130 | 69 |  | -1 |
| tRNA^Met^ | H | 130 | 198 | 69 |  | 0 |
| NAD2 | H | 199 | 1201 | 1003 | 334* | 0 |
| tRNA^Trp^ | H | 1202 | 1269 | 68 |  | 0 |
| tRNA^Cys^ | L | 1262 | 1328 | 67 |  | -8 |
| tRNA^Tyr^ | L | 1331 | 1397 | 67 |  | 1 |
| COI | H | 1399 | 2932 | 1534 | 511* | 0 |
| tRNA^Leu2^ | H | 2933 | 2997 | 65 |  | 0 |
| COII | H | 2998 | 3676 | 679 | 226* | 0 |
| tRNA^Lys^ | H | 3677 | 3748 | 72 |  | -1 |
| tRNA^Asp^ | H | 3748 | 3811 | 64 |  | 0 |
| ATP8 | H | 3812 | 3967 | 156 | 52 | -7 |
| ATP6 | H | 3961 | 4627 | 667 | 222* | 0 |
| COIII | H | 4628 | 5414 | 787 | 262* | 0 |
| tRNA^Gly^ | H | 5415 | 5477 | 63 |  | 0 |
| NAD3 | H | 5478 | 5831 | 354 | 118 | 0 |
| tRNA^Ala^ | H | 5832 | 5893 | 62 |  | 1 |
| tRNA^Arg^ | H | 5895 | 5961 | 67 |  | -1 |
| tRNA^Asn^ | H | 5961 | 6027 | 67 |  | 0 |
| tRNA^Ser1^ | H | 6028 | 6097 | 70 |  | -1 |
| tRNA^Glu^ | H | 6097 | 6163 | 67 |  | -1 |
| tRNA^Phe^ | L | 6163 | 6229 | 67 |  | 0 |
| NAD5 | L | 6230 | 7931 | 1702 | 567* | 1 |
| tRNA^His^ | L | 7933 | 7995 | 63 |  | 1 |
| NAD4 | L | 7997 | 9328 | 1332 | 444 | -5 |
| NAD4L | L | 9324 | 9627 | 304 | 101* | 2 |
| tRNA^Thr^ | H | 9630 | 9692 | 63 |  | 0 |
| tRNA^Pro^ | L | 9693 | 9755 | 63 |  | 7 |
| NAD6 | H | 9762 | 10262 | 501 | 167 | -1 |
| COB | H | 10262 | 11396 | 1135 | 378* | 0 |
| tRNA^Ser2^ | H | 11397 | 11465 | 69 |  | 20 |
| NAD1 | L | 11486 | 12406 | 921 | 307 | 0 |
| tRNA^Leu1^ | L | 12407 | 12471 | 65 |  | 0 |
| 16S rRNA | L | 12472 | 13736 | 1265 |  | 0 |
| tRNA^Val^ | L | 13737 | 13805 | 69 |  | 2 |
| 12S rRNA | L | 13808 | 14567 | 760 |  | 0 |
| ORF | H | 14568 | 15281 | 714 |  | 0 |

Asterisks (*) indicate proteins with incomplete stop codons.
