# Supplementary material for: The discovery of an overseen pygmy backswimmer in Europe (Heteroptera, Nepomorpha, Pleidae)
Source: Sci Rep. 2024 Nov 15;14:28139. doi: 10.1038/s41598-024-78224-6 (PMC11568165; doi:10.1038/s41598-024-78224-6)
Supplement: Supplementary file 7 — Supplementary Material 7 [file 41598_2024_78224_MOESM7_ESM.docx]

**Supplementary Table S5:** Summary of the analyzed genes, alignment lengths, and substitution models used in the phylogenomic analysis of the Notonectoidea based on mitochondrial genomes.

| **Gene** | **Alignment length [base pairs]** | **Substitution model** |
| --- | --- | --- |
| atp6 | 714 | TIM+F+I+G4 |
| atp8 | 159 | TPM2+F+I+G4 |
| cox1 | 1,539 | GTR+F+I+G4 |
| cox2 | 679 | TIM+F+I |
| cox3 | 787 | GTR+F+R2 |
| cytb | 1,137 | GTR+F+I+G4 |
| nad1 | 942 | TIM+F+G4 |
| nad2 | 1,014 | GTR+F+I+G4 |
| nad3 | 357 | TIM+F+I+G4 |
| nad4 | 1,338 | TIM+F+I+G4 |
| nad4l | 315 | K3Pu+F+G4 |
| nad5 | 1,710 | TIM+F+I+G4 |
| nad6 | 510 | TN+F+I+G4 |
| 12S rDNA | 750 | GTR+F+G4 |
| 16S rDNA | 1,221 | TVM+F+I+G4 |
