# Supplementary material for: The discovery of an overseen pygmy backswimmer in Europe (Heteroptera, Nepomorpha, Pleidae)
Source: Sci Rep. 2024 Nov 15;14:28139. doi: 10.1038/s41598-024-78224-6 (PMC11568165; doi:10.1038/s41598-024-78224-6)
Supplement: Supplementary file 10 — Supplementary Material 10 [file 41598_2024_78224_MOESM10_ESM.docx]

**Supplementary information**

**Supplementary Movie S1:** Movie based on the 3D scan of *Plea m. minutissima* Leach, 1817 (specimen from Germany, Haltern, Flaesheim) using Adobe Premiere Pro 24.3.0 (Adobe Systems Software Ireland Limited, Dublin, Ireland).

**Supplementary Movie S2:** Movie based on the 3D scan of *Plea cryptica* sp. nov. (specimen from Germany, Brandenburg, Havelland, Götzerberge) using Adobe Premiere Pro 24.3.0 (Adobe Systems Software Ireland Limited, Dublin, Ireland).
